# Investigating regulatory patterns of NLRP3 Inflammasome features and association with immune microenvironment in Crohn’s disease

**DOI:** 10.3389/fimmu.2022.1096587

**Published:** 2023-01-05

**Authors:** Huihuan Wu, Ruijie Zeng, Xinqi Qiu, Kequan Chen, Zewei Zhuo, Kehang Guo, Yawen Xiang, Qi Yang, Rui Jiang, Felix W. Leung, Qizhou Lian, Weihong Sha, Hao Chen

**Affiliations:** ^1^ Department of Gastroenterology, Guangdong Provincial People’s Hospital, Guangdong Academy of Medical Sciences, Guangzhou, China; ^2^ School of Medicine, South China University of Technology, Guangzhou, China; ^3^ School of Medicine, Shantou University Medical College, Shantou, China; ^4^ Zhuguang Community Healthcare Center, Guangzhou, China; ^5^ Department of Gastroenterology, The First Affiliated Hospital of Guangzhou Medical University, Guangzhou, China; ^6^ Department of Critical Care Medicine, The Fifth Affiliated Hospital of Zhengzhou University, Zhengzhou, China; ^7^ Edinburgh Medical School, College of Medicine and Veterinary Medicine, University of Edinburgh, Edinburgh, United Kingdom; ^8^ David Geffen School of Medicine, University of California Los Angeles, Los Angeles, CA, United States; ^9^ Department of Medicine, Queen Mary Hospital, Hong Kong, Hong Kong SAR, China

**Keywords:** Crohn’s disease, NLRP3 inflammasome, immune landscape, drug, treatment

## Abstract

**Introduction:**

Crohn’s disease is characterized of dysregulated inflammatory and immune reactions. The role of the NOD-like receptor family, pyrin domain-containing 3 (NLRP3) inflammasome in Crohn’s disease remains largely unknown.

**Methods:**

The microarray-based transcriptomic data and corresponding clinical information of GSE100833 and GSE16879 were obtained from the Gene Expression Omnibus (GEO) database. Identification of in the NLRP3 inflammasome-related genes and construction of LASSO regression model. Immune landscape analysis was evaluated with ssGSEA. Classification of Crohn’s-disease samples based on NLRP3 inflammasome-related genes with ConsensusClusterPlus. Functional enrichment analysis, gene set variation analysis (GSVA) and drug-gene interaction network.

**Results:**

The expressions of NLRP3 inflammasome-related genes were increased in diseased tissues, and higher expressions of NLRP3 inflammasome-related genes were correlated with generally enhanced immune cell infiltration, immune-related pathways and human leukocyte antigen (HLA)-gene expressions. The gene-based signature showed well performance in the diagnosis of Crohn’s disease. Moreover, consensus clustering identified two Crohn’s disease clusters based on NLRP3 inflammasome-related genes, and cluster 2 was with higher expressions of the genes. Cluster 2 demonstrated upregulated activities of immune environment in Crohn’s disease. Furthermore, four key hub genes were identified and potential drugs were explored for the treatment of Crohn’s disease.

**Conclusions:**

Our findings indicate that NLRP3 inflammasome and its related genes could regulate immune cells and responses, as well as involve in the pathogenesis of Crohn’s disease from transcriptomic aspects. These findings provide in silico insights into the diagnosis and treatment of Crohn’s disease and might assist in the clinical decision-making process.

## 1 Introduction

Crohn’s disease is one of the two major forms of inflammatory bowel diseases (IBD) that lead to relapsing and remitting inflammation of the digestive tract (1). It is estimated that over 2 million and 1.5 million people suffer from IBD in Europe and North America, respectively (2). Although the incidence of IBD is relatively stable in western countries, in newly industrialized countries with more westernized societies, the incidence of Crohn’s disease is still increasing (2). Despite treatment advances, Crohn’s disease is with high morbidity and substantial healthcare costs (1, 3).

In the past two decades, substantial progress has been achieved in the field of treatment for Crohn’s disease (4). The advanced understating of the inflammatory cascades, cytokines and adhesion molecules essential in the pathogenesis of Crohn’s diseases has largely promoted the pharmacological armamentarium (5). For example, the anti-tumor necrosis factor (TNF) drug infliximab is effective for treating Crohn’s disease with a good safety profile (6). However, a significant proportion of the patients demonstrate no clinical benefit from biological agents, and the response could be lost over time (5, 6). Identifying new targets and developing novel therapeutics is essential to assist in the treatment of Crohn’s disease.

The etiology of Crohn’s disease remains largely unknown, and consequently, clarifying the mechanisms of pathogenesis is important. Inflammasomes are well-known to involve in inflammatory disorders in various systems (7). The NOD-like receptor family, pyrin domain-containing 3 (NLRP3) inflammasome, which serves as an intracellular sensor for detecting a broad range of signals, is most extensively studied among all types of inflammasomes (8). NLRP3 inflammasome was reported to participate in the pathogenesis, progression and treatment response of Crohn’s disease (9). However, a comprehensive overview of NLRP3 inflammasome and its related molecules in Crohn’s disease is lacking.

In this study, we aimed to provide a comprehensive overview of NLRP3 inflammasome-related genes and pathways in Crohn’s disease by a computational approach. Machine learning was applied to classify the patients based on the NLRP3 inflammasome-related genes. The correlations with immune landscape and potential drugs were further investigated.

## 2 Materials and methods

### 2.1 Dataset and NLRP3 inflammasome-related gene set

The microarray data and corresponding clinical information of GSE100833 and GSE16879 were obtained from the Gene Expression Omnibus (GEO) database. In total, the samples obtained from dataset GSE100833 includes 159 samples derived from inflamed areas of CD patient undergoing surgical bowel resection, and 168 samples derived from normal, non-inflamed bowel located more than 10 cm away from patients undergoing surgical bowel resection for sporadic colon cancer, and the samples with Crohn’s disease were all isolated from areas with moderate to severe inflammation (10). The samples obtained from the dataset GSE16879 include 12 samples derived from healthy individuals undergoing endoscopic biopsy for polyp screening, and 73 samples derived from active CD patients undergoing endoscopic biopsy (11). **Supplementary Table 1** summarizes the available clinicopathological characteristics of each sample. The 30 *homo sapiens* NLRP3 inflammasome-related genes were acquired from the publication by Ju et al. (12). Briefly, the genes were defined according to the Gene Ontology (GO) (GO: 0044546, GO: 0072559) and Reactome (R-HSA-844456).

### 2.2 Protein-protein interaction network and chromosome location

Genomic locations of the 30 NLRP3 inflammasome-related genes in chromosomes were demonstrated using Circos (13). PPI networks of the NLRP3 inflammasome-related genes were created from the Search Tool for the Retrieval of Interacting Genes/Proteins (STRING) database (https://string-db.org), and an interaction score > 0.4 was considered as statistically significant, and hiding individual target protein nodes (14). The data obtained from the String database were input into Cytoscape3.8.0 to visualize the PPI network.

### 2.3 Identification of differentially expressed genes in the NLRP3 inflammasome-related genes and construction of LASSO regression model

For the GSE100833 data and GSE16879 data, we first used Wilcoxon test to analyze the expression of NLRP3 inflammasome-related genes between the diseased samples and normal tissues. The DEGs were further identified by the limma package (15) with the threshold of adjusted *P* < 0.05 between the diseased samples and normal tissues. Volcano plots and heatmaps were used to demonstrate the expressions of DEGs between the two groups. The DEGs that were associated with Crohn’s disease development were further sorted by univariate logistic regression analysis. LASSO cox regression was performed by the glmnet package to remove redundant genes and construct a refined model (16). Receiver operating characteristic (ROC) curve and calculation of the area under the curve (AUC) was performed in PRISM 8.0.

### 2.4 Immune landscape analysis

Single-sample gene set enrichment analysis (ssGSEA) was performed by the GSVA package (17) to assess the differences in immune landscape. The infiltrations of immune cells (24 types), the alternations of immune-related pathways, and the expressions of HLA genes were evaluated between the two groups.

### 2.5 Classification of Crohn’s-disease samples based on NLRP3 inflammasome-related genes

To classify the Crohn’s disease samples into two groups, the ConsensusClusterPlus package was used for cluster analysis (distance = “Euclidean”; clusterAlg = “km”; reps = 100) (18). The DEGs between the two subgroups were identified by the limma package (adjusted *P* < 0.05, |log2FC| > 1) (15).

### 2.6 Functional enrichment analysis, gene set variation analysis and drug-gene interaction network

Based on the DEGs, the GO and Kyoto Encyclopedia of Genes and Genomes (KEGG) pathway enrichment analyses between the two subgroups were performed using the clusterProfiler package (19). GSVA was also performed to elucidate the machinimas by the GSVA package (17). PPIs with STRING score > 900 were used to construct the interaction network and visualized by cytoscape (20). The Drug–Gene Interaction Database (DGIdb) database serves as a central repository for data on drug-gene interactions and druggability gathered from various sources, providing information on drug-gene interactions and druggable genes (21). The drug-gene interaction network was explored by the DGIdb after excluding all non-specific drugs that targeted > 10 genes from the analysis (21).

### 2.7 Statistical analysis

Statistical analysis was performed by R (version 4.1.0) and SPSS (version 25.0) software. T-test or Wilcoxon rank-sum test was used to analyze the continuous variables according to the normality. Pearson chi-square test was used to examine the differences of categorical variables. All significant thresholds were set at a two-sided *P* < 0.05.

## 3 Results

### 3.1 Location and PPIs of NLRP3 inflammasome-related genes

**Figure 1:**
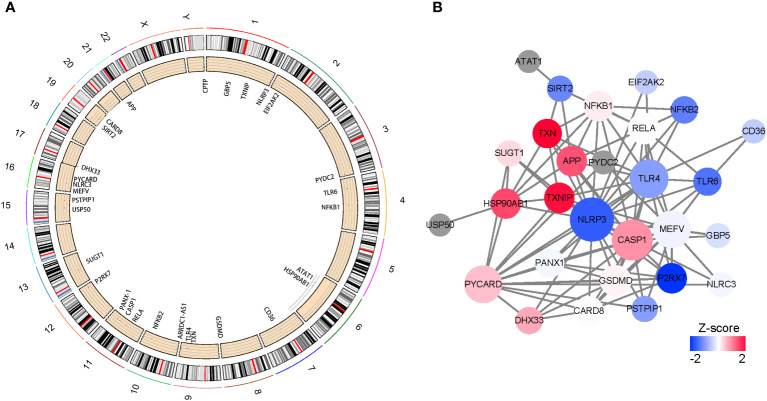
Characteristics and interactions of 25 NLRP3 inflammasome-related genes. **(A)** Circos plot showing the location of genes in 24 chromosomes. **(B)** protein-protein interaction networks of DEGs. Red represents positive correlations while blue represents negative correlations.

The locations of 30 NLRP3 inflammasome-related genes, including *ARRDC1-AS1, CARD8, GSDMD, ATAT1,CD36,CPTP,DHX33,EIF2AK2,GBP5,NLRC3,PYDC2,SIRT2,TLR4,TLR6,USP50,APP,CASP1,HSP90AB1,MEFV,NFKB1,NFKB2,NLRP3,P2RX7,PANX1,PSTPIP1,PYCARD,RELA,SUGT1,TXN,TXNIP*, were demonstrated in **Figure 1A**. PPIs exist among the inflammasome-related genes, in which NLRP3, Toll-like receptor 4 (TLR4) and Caspase 1 (CASP1) were with higher numbers of interacted proteins, and different colors reflect different correlation coefficient.(**Figure 1B**).

### 3.2 Differentially expressed NLRP3 inflammasome-related genes

**Figure 2:**
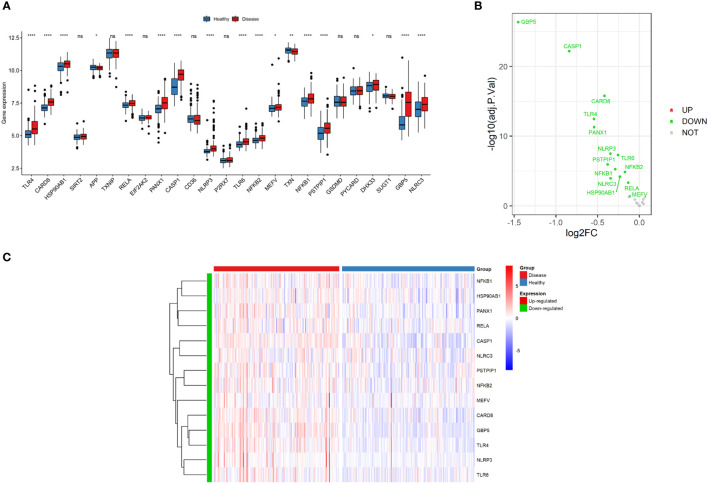
Expressions of NLRP3 inflammasome-related genes. **(A)** Box plot demonstrating the overview of gene expressions in the GSE100833 dataset. Blue: healthy (non-inflamed) tissues; Red: diseased (inflamed) tissues. **(B)** Volcano plot illustrating the differentially expressed genes (DEGs) in the GSE100833 dataset. Green: downregulation. Red: upregulation. Grey: not significantly altered. **(C)** Heatmap for the DEGs in the GSE100833 dataset. Blue: tissues from healthy control; Red: tissues from patients with Crohn’s disease. ns: *P* ≥ 0.05; *: *P* < 0.05; ** *P* ≤ 0.01; **** *P* ≤ 0.0001.

A total of 30 NLRP3 inflammasome-related genes were included in our study. However, there are 5 NLRP3 inflammasome-related genes for which some expression data is lacking. As a result, 25 NLRP3 genes having expression data were chosen for further analysis, and 14 DEGs were identified (*CARD8, CASP1, GBP5, HSP90AB1, MEFV, NFKB1, NFKB2, NLRC3, NLRP3, PANX1, PSTPIP1, RELA, TLR4* and *TLR6*) in the inflamed samples compared to non-inflamed samples in GSE100833 (**Figure 2A** **;** **Supplementary Table 2**). All of the DEGs were downregulated in the non-inflamed tissues (**Figures 2B, C** **)**.

### 3.3 Correlation of NLRP3 inflammasome-related genes

**Figure 3:**
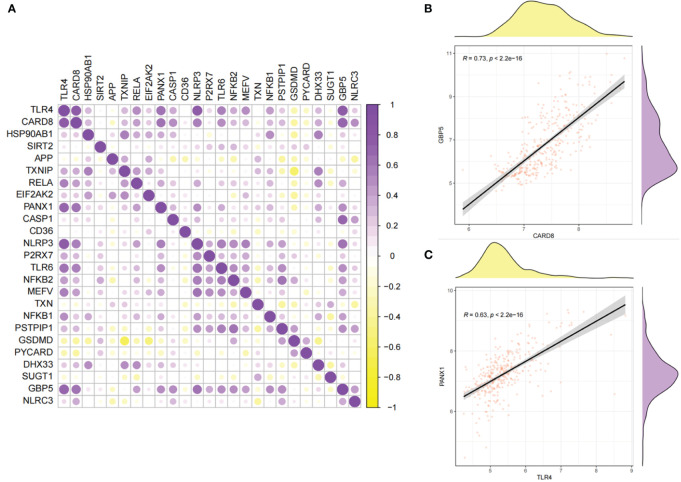
Correlations between the expressions of 25 NLRP3 inflammasome-related genes. **(A)** Correlation plot showing significant correlations between the genes. Purple: positive correlation. Yellow: negative correlation. Circles with deeper colors indicate stronger correlations. **(B)** Correlation between *CARD8* and *GBP5* expressions in integrated samples. **(C)** Correlation between *TLR4* and *PANX1* expressions in diseased samples.

The correlations of 25 NLRP3 inflammasome-related genes were further explored in the diseased and normal samples (**Figure 3A**). In the integrated diseased and normal samples, strong positive correlation was observed (r = 0.73, *P* < 2.2e-16) between *CARD8* and *GBP5* expressions (**Figure 3B**). For the diseased samples, strong positive correlation was observed (r = 0.63, *P* < 2.2e-16) between *TLR4* and *PANX1* expressions (**Figure 3C**).

### 3.4 Construction of prediction model for Crohn’s disease

**Figure 4:**
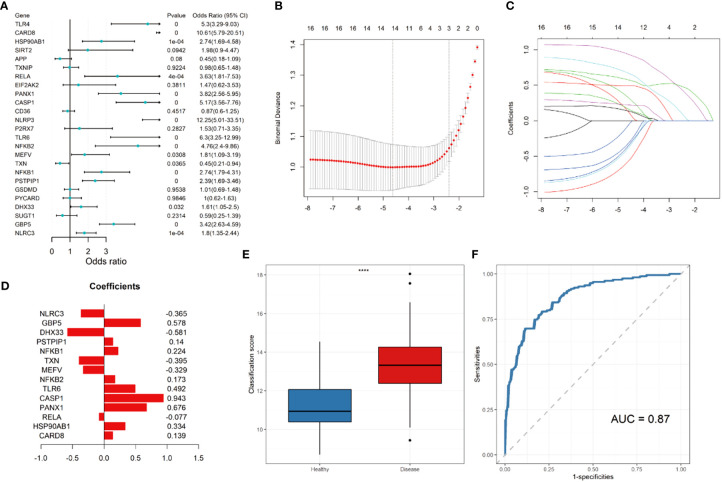
Construction of LASSO regression model and receiver operating characteristic (ROC) curve. **(A)** Forest plot showing the odds ratio and 95% confidence intervals for predicting Crohn’s disease of each gene. **(B)** Log lambda values of the genes corresponding to the minimum cross-validation error point. **(C)** Selection of genes with non-zero coefficient for construction of model. **(D)** Coefficients of each gene within the prediction model. **(E)** Box plot showing the scores of in the disease (red) and control group (blue). **(F)** ROC curve for prediction of Crohn’s disease. **** *P* ≤ 0.0001.

Univariate logistic regression demonstrated that 16 DEGs were associated with Crohn’s disease (**Figure 4A**). LASSO regression analysis was performed for further selecting the predictive genes, and 14 of the 16 genes were included in the prediction model (**Figures 4B, C** **)**. The coefficient of each gene was demonstrated in **Figure 4D**. According to **Figure 4E**, the diseased samples possess significantly higher scores calculated by the prediction model. The ROC curve indicates that the AUC of the prediction model was 0.87 (**Figure 4F**).

### 3.5 NLRP3 inflammasome-related genes are associated with alterations in immune landscape

**Figure 5:**
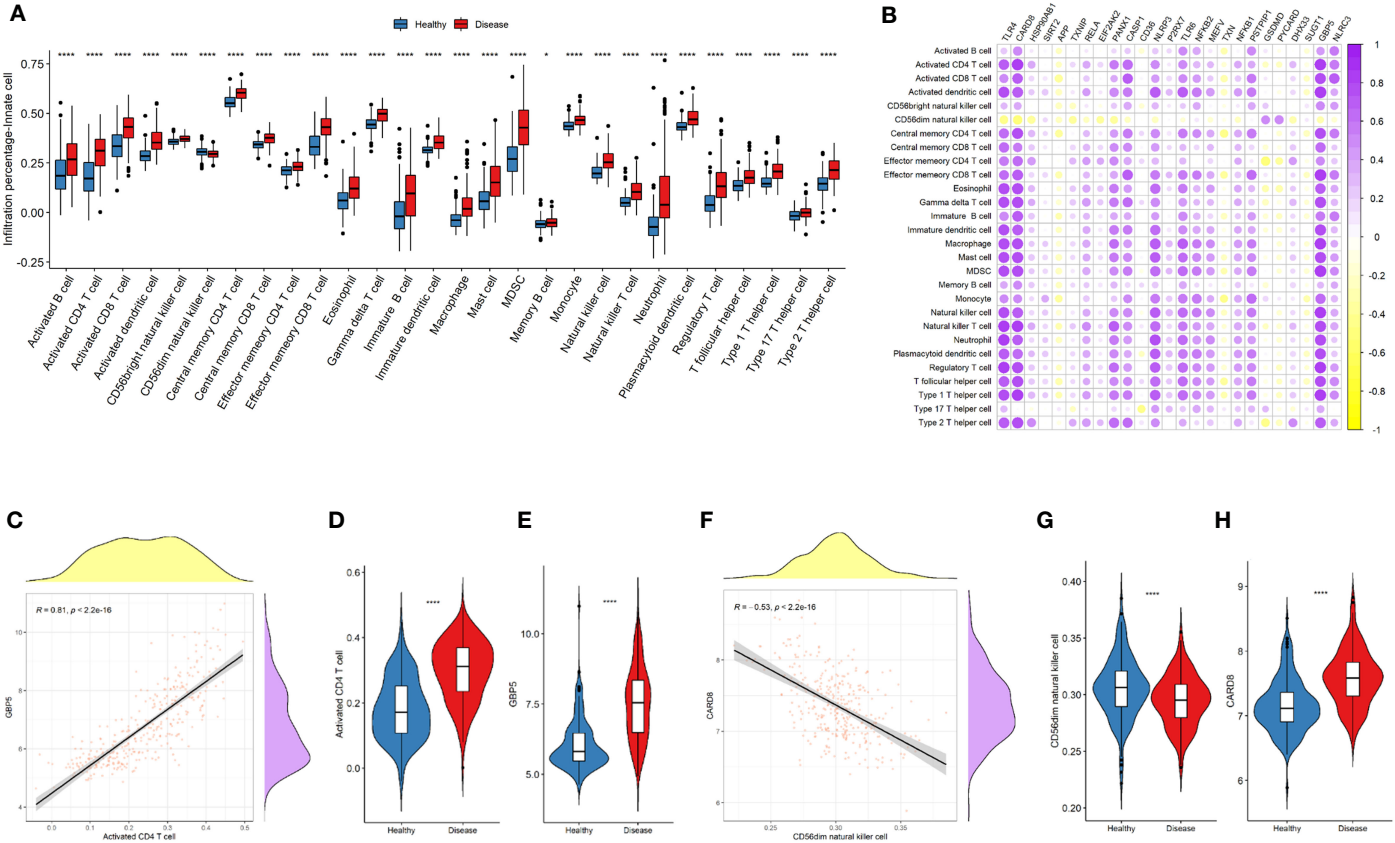
NLRP3 inflammasome-related genes and immune cell infiltrations in the immune landscape. **(A)** Comparison of immune cell infiltration levels between the two groups. **(B)** Correlation plot showing significant correlations between the genes and immune cell infiltrations. Purple: positive correlation. Yellow: negative correlation. Circles with deeper colors indicate stronger correlations. **(C)** Correlation between GBP5 expression and activated CD4^+^ T cell infiltration. **(D, E)** Violin plots showing the levels of activated CD4^+^ T cell **(D)** and GBP5 expression **(E)** in two groups. **(F)** Correlation between CARD8 expression and CD56dim natural killer cell infiltration. **(G, H)** Violin plots showing the levels of CD56dim natural killer cell **(G)** and CARD8 expression **(H)** in two groups. *R* represents Pearson correlation coefficients. *: *P* < 0.05; **** *P* ≤ 0.0001.

To further investigate the differences in the immune landscape, as well as the correlations between NLRP3 inflammasome-related genes and immune cell infiltration, ssGSEA analysis was performed. Nearly all types of immune cells were significantly enriched in the microenvironment of disease samples (**Figure 5A**). Higher expressions of most of the NLRP3 inflammasome-related genes were significantly correlated to the increased infiltration of immune cells (**Figure 5B**). The positive correlation between *GBP5* expressions and infiltrations of activated CD4^+^ T cells was most significant (r = 0.81, *P* < 2.2e-16; **Figures 5C–E**). The negative correlation between *CARD8* expressions and CD56dim-natural-killer-cell infiltration was most significant (r = -0.53, *P* < 2.2e-16; **Figures 5F–H**).

**Figure 6:**
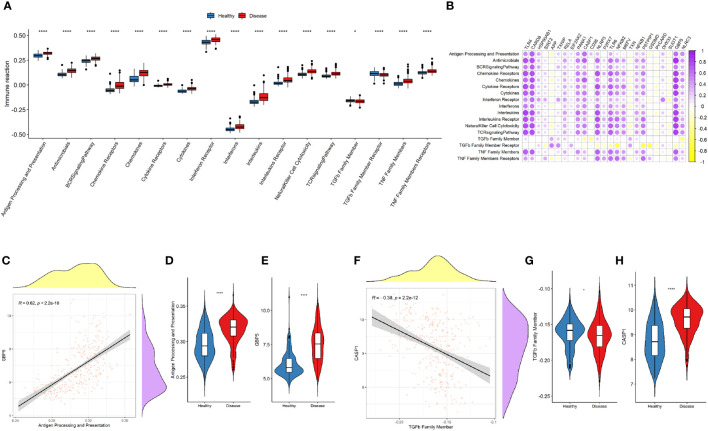
NLRP3 inflammasome-related genes and immune-related pathways. **(A)** Comparison of immune-related pathway activations between the two groups. **(B)** Correlation plot showing significant correlations between the genes and immune-related pathways. Purple: positive correlation. Yellow: negative correlation. Circles with deeper colors indicate stronger correlations. **(C)** Correlation between GBP5 expression and antigen processing and presentation pathway. **(D, E)** Violin plots showing the levels of antigen processing and presentation pathway **(D)** and GBP5 expression **(E)** in two groups. **(F)** Correlation between CASP1 expression and TGFβ family member. **(G, H)** Violin plots showing the levels of TGFβ family member **(G)** and CASP1 expression **(H)** in two groups. *R* represents Pearson correlation coefficients. *: *P* < 0.05; **** *P* ≤ 0.0001.

The immune-related pathways were generally upregulated in the disease samples (**Figure 6A**). The upregulation of most of the NLRP3 inflammasome-related genes was significantly related to the increased activity of immune-related pathways (**Figure 6B**). Among the NLRP3 inflammasome-related genes, *GBP5* was most significantly associated with the activation of antigen processing and presentation pathway (r = 0.62, *P* < 2.2e-16; **Figures 6C–E**). *CASP1* was most significantly correlated with the downregulation of TGFb family members (r = -0.38, *P* = 2.2e-12; **Figures 6F–H**).

**Figure 7:**
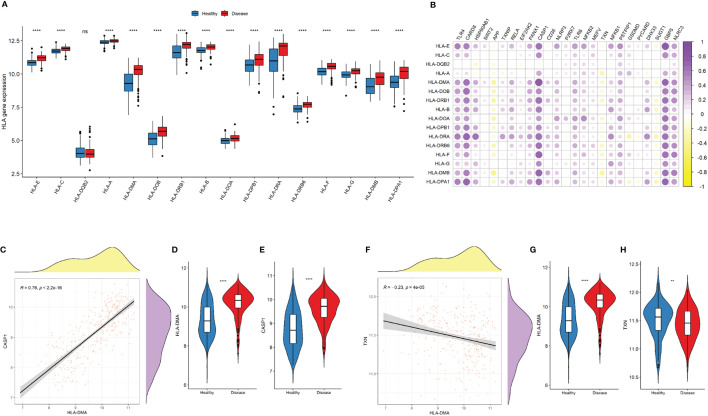
NLRP3 inflammasome-related genes and HLA-related genes. **(A)** Comparison of HLA-related gene expressions between the two groups. **(B)** Correlation plot showing significant correlations between the NLRP3 inflammasome- and HLA-related genes. Purple: positive correlation. Yellow: negative correlation. Circles with deeper colors indicate stronger correlations. **(C)** Correlation between CASP1 expression and antigen processing and presentation pathway. **(D, E)** Violin plots showing the expressions of HLA-DMA **(D)** and CASP1 expression **(E)** in two groups. **(F)** Correlation between TXN and HLA-DMA expressions **(G-H)** Violin plots showing the expressions of HLA-DMA **(G)** and TXN **(H)** in two groups. *R* represents Pearson correlation coefficients. ns: P ≥ 0.05; ** P ≤ 0.01; **** P ≤ 0.0001.

Expressions of most of the human leukocyte antigen (HLA)-related genes were increased in the disease group (**Figure 7A**). The majority of NLRP3 inflammasome-related genes were significantly correlated with increased expressions of HLA-related genes (**Figure 7B**). *CASP1* was most significantly correlated with the upregulation of HLA-DMA (r = 0.76, *P* < 2.2e-16; **Figures 7C–E**). By contrast, *TXN* was most significantly correlated with the downregulation of TGFb family members (r = -0.23, *P* = 4e-05; **Figures 7F–H**).

### 3.6 Identification of two Crohn’s disease subtypes based on NLRP3 inflammasome-related gene

**Figure 8:**
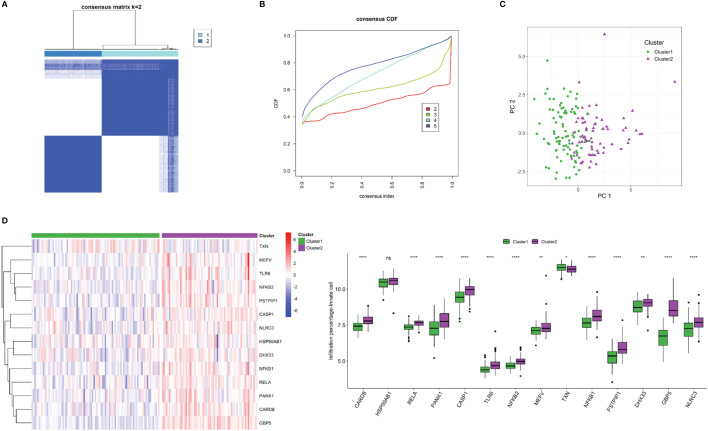
NLRP3 inflammasome-related gene-based classification of Crohn’s disease. **(A)** Unsupervised consensus clustering matrix and optimal clusters. **(B)** Item-consensus plot showing the relationship between each cluster. **(C)** Principal component analysis (PCA) based on clustering results. **(D)** Heatmap and box plots demonstrating expressions of NLRP3 inflammasome-related genes in each cluster. ns: *P* ≥ 0.05; *: *P* < 0.05; ** *P* ≤ 0.01; **** *P* ≤ 0.0001.

Using unsupervised *K*-mean consensus clustering, we identified two subtypes of Crohn’s disease based on the 14 NLRP3 inflammasome-related genes. The optimal clusters were derived when *K* = 2 (**Figures 8A, B** **)**. Principal component analysis (PCA) demonstrated good distinction of the two clusters (**Figure 8C**). The 14 NLRP3 inflammasome-related genes were generally upregulated in cluster 2 compared to cluster 1, except for *TXN* (**Figure 8D**). The clinicopathological characteristics of the two clusters were shown in **Supplementary Figure 1**. The distributions of tissue location, age, and gender were not significantly different (**Supplementary Figure 1**).

### 3.7 NLRP3 inflammasome-related gene-based clusters were associated with immune landscape

**Figure 9:**
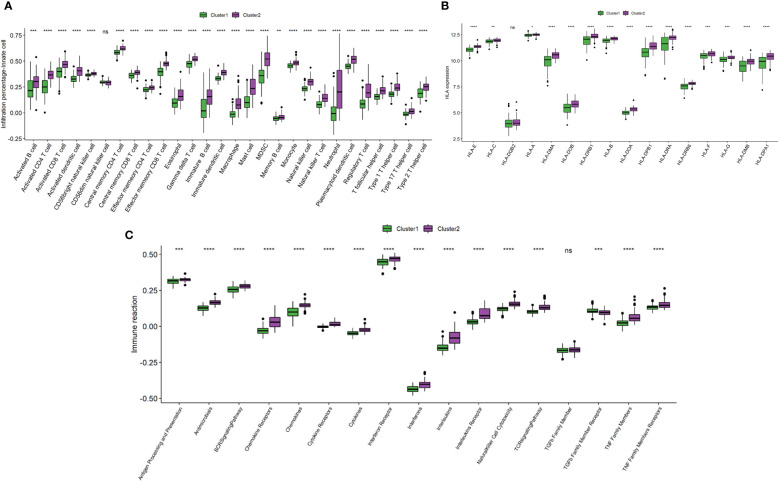
Comparison of immune microenvironment between clusters. **(A)** Box plot showing immune cell infiltration levels in two clusters. **(B)** Box plot demonstrating HLA expressions in two clusters. **(C)** Box plot illustrating immune reaction in two clusters. ns: *P* ≥ 0.05; *: *P* < 0.05; ** *P* ≤ 0.01; *** *P* ≤ 0.001; **** *P* ≤ 0.0001.

To investigate the differences in immune landscape between clusters, ssGSEA was performed. The infiltration levels of immune cells were generally higher in cluster 2 than in cluster 1 (**Figure 9A**). Similarly, cluster 2 is with higher HLA expressions (**Figure 9B**) and immune-related pathways (**Figure 9C**) than cluster 1.

### 3.8 Pathway enrichment analysis and GSVA revealed enriched pathways in clusters

**Figure 10:**
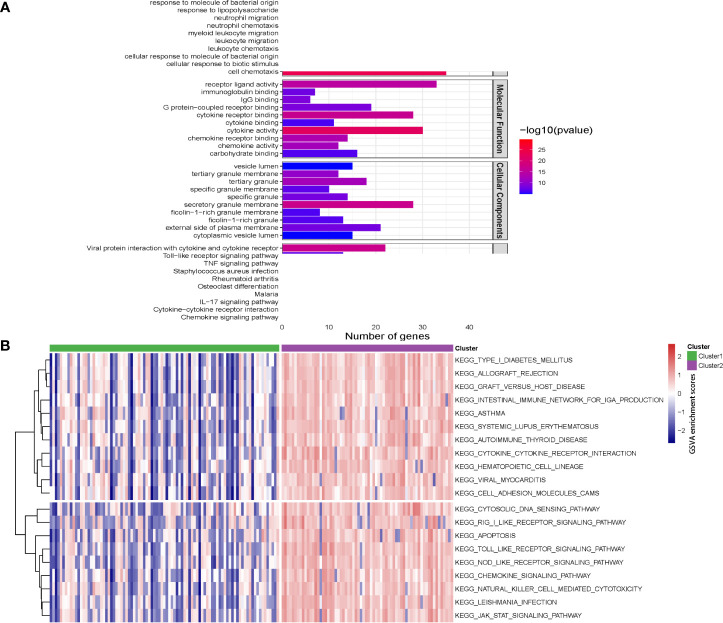
Pathway enrichment analysis and gene set variation analysis (GSVA) for two Crohn’s disease clusters. **(A)** The top 10 enriched gene sets by GO pathway (biological process, molecular function and cellular components) and KEGG pathway analysis according to enrichment scores. **(B)** Heatmap showing enriched pathways for single hub gene through GSVA.

Pathway enrichment analysis and GSVA were performed to elucidate the function of genes of the two clusters. As indicated by **Figure 10A**, GO pathway analysis revealed that immune-related pathways, including neutrophil regulation, leukocyte regulation, cytokine activity, were upregulated in cluster 2. KEGG pathway analysis demonstrated that the TLR-, TNF-, IL-17- and chemokine-signaling pathways were activated in cluster 2 (**Figure 10A**). GSVA analysis revealed the 77 differences in signaling pathways by cluster 1 vs cluster 2 (**Figure 10B**).

### 3.9 Prediction of drug-gene interaction and identification of potential drugs

**Figure 11:**
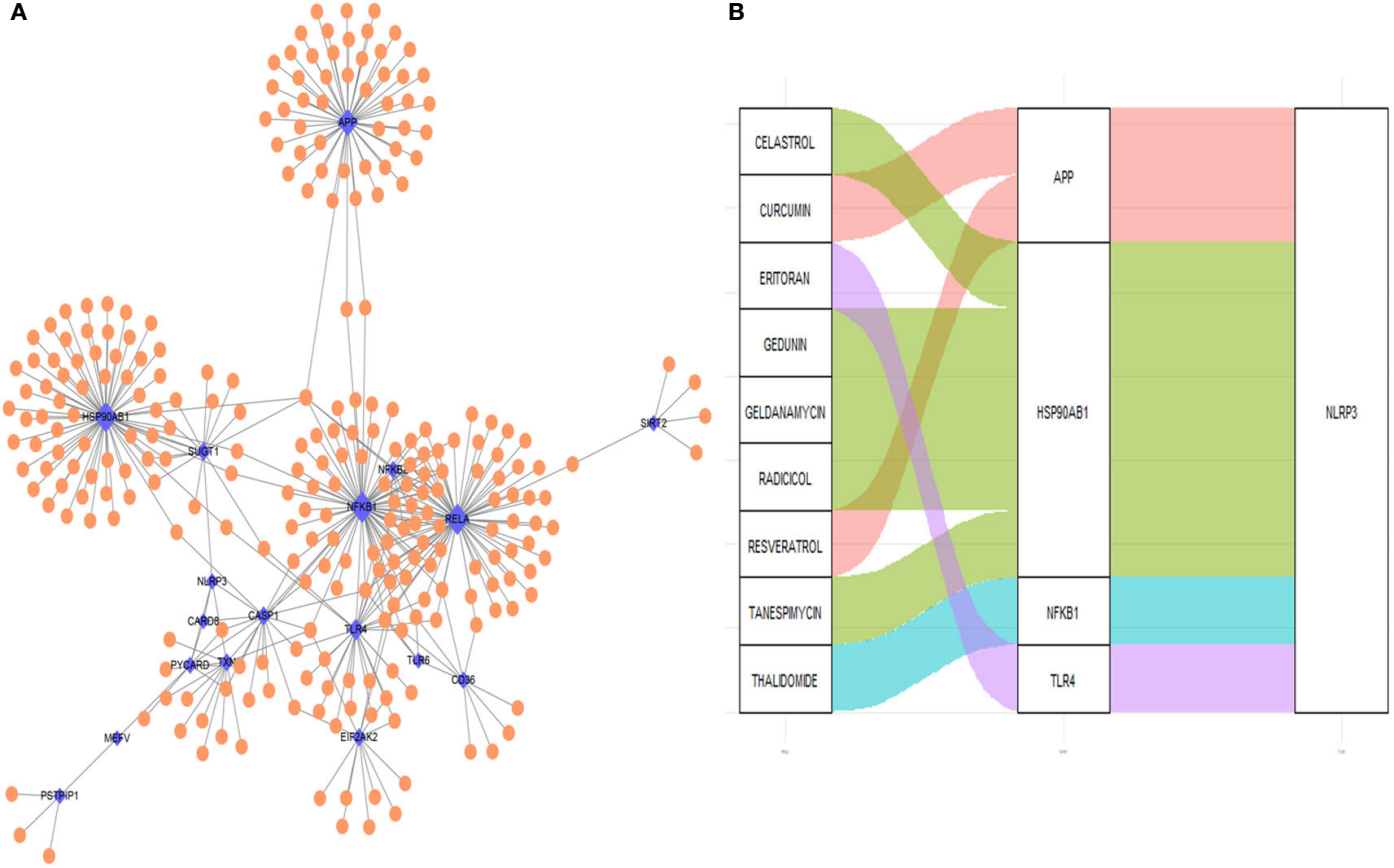
Drug-gene interaction network and potential drug prediction. **(A)** Network of protein-protein interaction. **(B)** Drug-interaction prediction of key genes. Four key genes (APP, HSP90AB1, NFKB1 and TLR4) were targeted in the DGIdb database. Nine potential target drugs were predicted from the database. Blue: NLRP3 inflammasome-related genes, orange: non-NLRP3 inflammasome-related genes. the size of the point indicate the drugs interacts with NLRP3 inflammasome-related genes.

The PPI network of the proteins of differentially expressed genes was demonstrated in **Figure 11A**. Nine NLRP3 inflammasome-related genes (*APP, CASP1, EIF2AK2, HSP90AB1, NFKB1, RELA, SUGT1, TLR4* and *TXN*) were further selected as the hub genes. Using the DGIdb database, four key NLRP3 inflammasome-related genes (*APP, HSP90AB1, NFKB* and *TLR4*) were further selected as potential druggable targets for Crohn’s disease treatment (**Figure 11B**). Two drugs targeted *APP*. Five drugs targeted *HSP90AB1*. One drug targeted *NFKB*, and one drug targeted *TLR4*.

## 4 Discussion

In the present study, we investigated and evaluated the role of NLRP3 inflammasome-related genes in the pathogenesis of Crohn’s disease. The expressions of NLRP3 inflammasome-related genes are generally upregulated in Crohn’s disease samples, and the gene-based signature could assist in the diagnosis of Crohn’s disease. Moreover, the immune landscape, including immune cell infiltration, immune response and HLA genes, are upregulated in diseased samples and could be regulated by NLRP3 inflammasome-related genes. Furthermore, two Crohn’s disease subtypes were identified by machine learning, and nine drugs could potentially target the hub genes of the subtypes.

Due to the multifactorial nature of Crohn’s disease, including genetic, environmental, and immunologic factors, its etiology remains largely unclear. It is deemed that Crohn’s disease is featured by inappropriate inflammatory and immune responses (22, 23). Inflammasomes, in which NLRP3 inflammasome is the most studied signaling platform, have been widely reported in various inflammation and immune disorders (24–27). The role of NLRP3 inflammasome in Crohn’s disease remains controversial, since its activation can protect intestinal epithelium by maintaining homeostasis, or aggravates intestinal damage *via* enhancing inflammation (28). Our study reveals that the significance of NLRP3 inflammasome-related genes in Crohn’s disease, and provide insights to disease development and progression.

To data, challenges still exist in the diagnosis of Crohn’s disease due to the polypharmacy and multimorbidity along with disease processes that mimic Crohn’s disease (29). In Asia, inadequate disease awareness, the lack of diagnostic services, the infectious diseases mimicking Crohn’s disease, and the extensively used antibiotics are critical challenges for diagnosing Crohn’s disease (30). Our model shows well performance in differentiating samples of Crohn’s disease from normal samples with AUC = 0.87, and therefore could assist in the clinical diagnosis of Crohn’s disease. Compared to the previously developed SNP-based (AUC = 0.86) (31) and matrix factorization-based machine learning model (AUC = 0.81) (32) by other researchers, our model demonstrates similar or better diagnostic values for Crohn’s disease.

The majority of the NLRP3 inflammasome-related genes are associated with enhanced infiltration and activation of immune cells and immune-related signaling. CD4^+^ T cells are known as the principal drivers of Crohn’s disease (33). B cells are critical for immune homeostasis, and B cell-related abnormalities are reported in patients with Crohn’s disease (34). Dendritic cells and macrophages initiate and maintain chronemic inflammation in Crohn’s disease (35). Neutrophils can produce reactive oxygen species and led to intestinal barrier damage in Crohn’s disease (36). Previous studies have also indicated the critical involvement of NLRP3 inflammasomes for the critical function of CD4^+^ T cell, B cells, dendritic cells and neutrophils, which confirm our results (37–40). In addition, immune-related pathways, including chemokines, cytokines, interleukins and TNF family members are activated by NLRP3 inflammasome-related genes. Moreover, most of the NLRP3 inflammasome-related genes are related to higher expressions of HLA genes, which indicate stronger immunogenicity. For instance, CARD8 is a potential candidate risk gene for CD, and it acts as a negative regulator of nuclear factor-kappa B as well as a suppressor of apoptosis. Previous research has indicated that CARD8 is up-regulated in the mucosa of Crohn’s disease patients (41, 42), which is consistent with our findings. Furthermore, our results mainly indicate that CARD8 might be involved in the regulation of the immune cell infiltration in Crohn’s disease, especially the CD56^dim^ subset of natural killer cells. Similarly, we have demonstrated the significantly positive correlation between CARD8 and GBP5 expressions. Further investigations on the pathogenesis of CD and how it affects the immune cell composition and GBP5 expression are warranted.

The concept of precision medicine has promoted the clustering of individual subjects. Different clusters have different pathogenic mechanisms and clinical prognostic characteristics. Similarly, different functional gene sets, such as immune-related and metabolism-related, were used to subgroup a single sample (43). In addition to the LASSO regression-based diagnostic model, unsupervised consensus clustering has identified two subtypes based on NLRP3 inflammasome-related genes. In this study, cluster 2 with higher expressions of NLRP3 inflammasome-related genes were significantly enriched with immune cells, activated immune reaction and upregulated HLA-gene expressions. Pathway enrichment analysis also revealed a number of immune-related pathways that were significantly activated in cluster 2. Taken together, our findings indicate that NLRP3 inflammasome-related genes are involved in the immune regulation of Crohn’s disease.

Although the role of the NLRP3 inflammasome in Crohn’s disease is debatable, our study reveals that the higher expressions of NLRP3 inflammasome-related genes were correlated with generally enhanced immune cell infiltration, immune-related pathways and human leukocyte antigen (HLA)-gene expressions. and the Crohn’s disease are associated with enhanced infiltration and activation of immune cells and immune-related signaling. Therefore, our findings indicate that NLRP3 inflammasome and its related genes could regulate immune cells and responses, as well as involve in the pathogenesis of Crohn’s disease.

Four key hub genes including *APP, HSP90AB1, NFKB1* and *TLR4* were identified. Amyloid-beta precursor protein (APP) is the precursor of amyloid β before sequential proteolytic cleavage (44), and amyloid β directly interacts with NLRP3 for the initiation of inflammasome activation (45). Heat shock protein 90kDa alpha, class B member 1 (HSP90AB1) is an isoform of the heat shock protein 90 (HSP90), and HSP90 inhibitor has been reported to inhibit NLRP3 inflammasome (46). Nuclear factor kappa B (NF-κB) drives the expression and activation of NLRP3 inflammasome (47, 48). Toll-like receptor 4 (TLR4) also mediates the activation of NLRP3 inflammasome (49). The four hub genes could account for the regulations and involvements of NLRP3 inflammasome in Crohn’s disease. Based on the interaction with the four hub genes, it is predicted that celastrol, curcumin, editoran, gedunin, geldanamycin, radicicol, resveratrol, tanespimycin and thalidomide are potential therapeutics that might be effective in treating Crohn’s disease.

Several limitations exist for our study. Our study mainly involves *in silico* data, and further studies based on cell and animal models could be performed in the future. In addition, current publicly available datasets are limited in the diversity of CD lesion locations, and therefore validation of different locations of the intestine in CD cohorts could be performed in future studies. Furthermore,the prognostic value and prediction of therapeutic responses based on the NLRP3 inflammasome-based signature could be further assessed with available information.

## 5 Conclusion

In conclusion, our study identified the dysregulations of NLRP3 inflammasome-related genes and their associations with the immune landscape, subtypes of patients, phenotype-related hub genes, and potential drugs of Crohn’s disease. Our findings provide a comprehensive overview of NLRP3 inflammasome-related genes in the etiology and promising possibilities for the treatment of Crohn’s disease. Further investigations on the specific mechanisms and validations are warranted.

## Data availability statement

The original contributions presented in the study are included in the article/**Supplementary Material**. Further inquiries can be directed to the corresponding authors.

## Author contributions

HW, RZ acquired the data, performed the analysis and wrote the manuscript. XQ and KC acquired the data and performed the analysis. ZZ, KG, YX, QY, and RJ participated in data analysis. FL, QL, WS and HC were involved in study design and supervision. FL, WS and HC acquired the funding. All authors contributed to the article and approved the submitted version.

## Supplementary material

Supplementary Figure 1 Clinicopathological characteristics of two clusters.
