# Supplementary figures and images for: Investigating regulatory patterns of NLRP3 Inflammasome features and association with immune microenvironment in Crohn’s disease
Source: Front Immunol. 2023 Jan 5;13:1096587. doi: 10.3389/fimmu.2022.1096587 (PMC9849378; doi:10.3389/fimmu.2022.1096587)

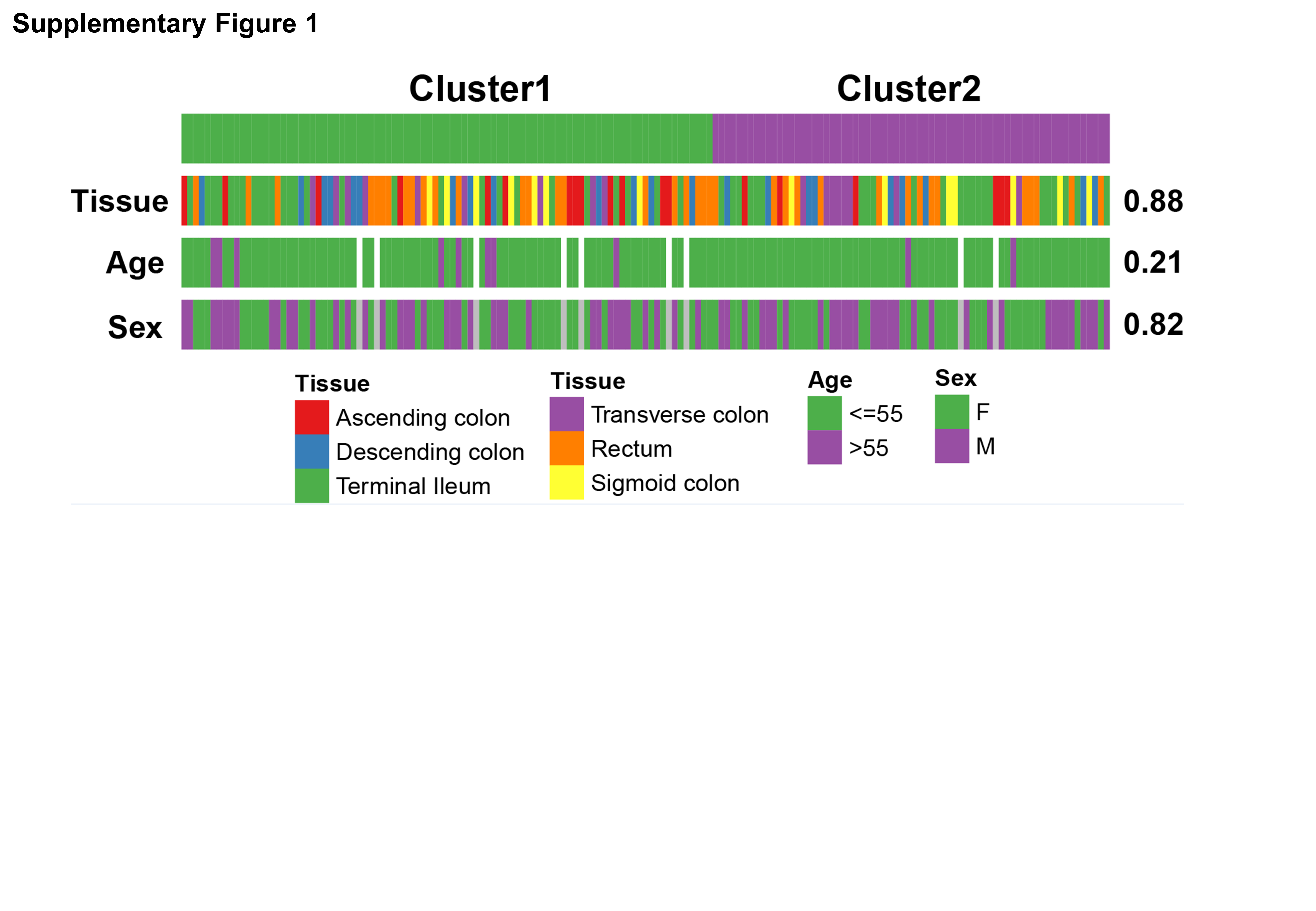

Supplement: Supplementary Figure 1 — Clinicopathological characteristics of two clusters. [file Image_1.tif]
