# Supplementary material for: Investigating regulatory patterns of NLRP3 Inflammasome features and association with immune microenvironment in Crohn’s disease
Source: Front Immunol. 2023 Jan 5;13:1096587. doi: 10.3389/fimmu.2022.1096587 (PMC9849378; doi:10.3389/fimmu.2022.1096587)
Supplement: Supplementary file 2 [file Table_1.docx]

**Supplementary Table 1a. Clinicopathological characteristics of the included samples in GSE100833 dataset.**

| **Source** | **Tissue /Location** | **Diagnosis** | **Age** | **Sex** | **inflammation** | **Disease activity** | **Method** |
| --- | --- | --- | --- | --- | --- | --- | --- |
| GSM2694187 | Ascending colon | Crohn's disease | 31 | M | Inflamed area | Moderate to severe | Surgical resection |
| GSM2694188 | Ascending colon | sporadic colon cancer | 51 | F | Non-involved area | normal tissue | Surgical resection |
| GSM2694189 | Rectum | sporadic colon cancer | 40 | M | Non-involved area | normal tissue | Surgical resection |
| GSM2694190 | Rectum | Crohn's disease | 52 | F | Inflamed area | Moderate to severe | Surgical resection |
| GSM2694191 | Terminal Ileum | Crohn's disease | 21 | M | Inflamed area | Moderate to severe | Surgical resection |
| GSM2694192 | Rectum | Crohn's disease | 44 | F | Inflamed area | Moderate to severe | Surgical resection |
| GSM2694193 | Rectum | sporadic colon cancer | 31 | M | Non-involved area | normal tissue | Surgical resection |
| GSM2694194 | Rectum | sporadic colon cancer | 34 | F | Non-involved area | normal tissue | Surgical resection |
| GSM2694195 | Descending colon | sporadic colon cancer | 26 | F | Non-involved area | normal tissue | Surgical resection |
| GSM2694196 | Descending colon | Crohn's disease | 38 | F | Inflamed area | Moderate to severe | Surgical resection |
| GSM2694197 | Rectum | sporadic colon cancer | 39 | F | Non-involved area | normal tissue | Surgical resection |
| GSM2694198 | Terminal Ileum | Crohn's disease | 36 | M | Inflamed area | Moderate to severe | Surgical resection |
| GSM2694199 | Ascending colon | sporadic colon cancer | 20 | F | Non-involved area | normal tissue | Surgical resection |
| GSM2694200 | Descending colon | Crohn's disease | 20 | M | Inflamed area | Moderate to severe | Surgical resection |
| GSM2694201 | Rectum | sporadic colon cancer | 63 | F | Non-involved area | normal tissue | Surgical resection |
| GSM2694202 | Terminal Ileum | Crohn's disease | 27 | F | Inflamed area | Moderate to severe | Surgical resection |
| GSM2694203 | Terminal Ileum | Crohn's disease | 55 | F | Inflamed area | Moderate to severe | Surgical resection |
| GSM2694204 | Terminal Ileum | Crohn's disease | 65 | M | Inflamed area | Moderate to severe | Surgical resection |
| GSM2694205 | Terminal Ileum | Crohn's disease | 60 | M | Inflamed area | Moderate to severe | Surgical resection |
| GSM2694206 | Terminal Ileum | sporadic colon cancer | 35 | M | Non-involved area | normal tissue | Surgical resection |
| GSM2694207 | Ascending colon | Crohn's disease | 35 | M | Inflamed area | Moderate to severe | Surgical resection |
| GSM2694208 | Terminal Ileum | Crohn's disease | 27 | M | Inflamed area | Moderate to severe | Surgical resection |
| GSM2694209 | Terminal Ileum | sporadic colon cancer | 48 | M | Non-involved area | normal tissue | Surgical resection |
| GSM2694210 | Ascending colon | sporadic colon cancer | 27 | M | Non-involved area | normal tissue | Surgical resection |
| GSM2694211 | Rectum | sporadic colon cancer | 51 | F | Non-involved area | normal tissue | Surgical resection |
| GSM2694212 | Terminal Ileum | sporadic colon cancer | 52 | F | Non-involved area | normal tissue | Surgical resection |
| GSM2694213 | Terminal Ileum | Crohn's disease | 35 | F | Inflamed area | Moderate to severe | Surgical resection |
| GSM2694214 | Terminal Ileum | Crohn's disease | 65 | M | Inflamed area | Moderate to severe | Surgical resection |
| GSM2694215 | Terminal Ileum | sporadic colon cancer | na | na | Non-involved area | normal tissue | Surgical resection |
| GSM2694216 | Ascending colon | sporadic colon cancer | 34 | M | Non-involved area | normal tissue | Surgical resection |
| GSM2694217 | Ascending colon | sporadic colon cancer | 28 | F | Non-involved area | normal tissue | Surgical resection |
| GSM2694218 | Terminal Ileum | Crohn's disease | 51 | F | Inflamed area | Moderate to severe | Surgical resection |
| GSM2694219 | Terminal Ileum | sporadic colon cancer | 42 | M | Non-involved area | normal tissue | Surgical resection |
| GSM2694220 | Terminal Ileum | sporadic colon cancer | 33 | M | Non-involved area | normal tissue | Surgical resection |
| GSM2694221 | Ascending colon | Crohn's disease | 26 | M | Inflamed area | Moderate to severe | Surgical resection |
| GSM2694222 | Terminal Ileum | sporadic colon cancer | 36 | F | Non-involved area | normal tissue | Surgical resection |
| GSM2694223 | Terminal Ileum | sporadic colon cancer | 26 | M | Non-involved area | normal tissue | Surgical resection |
| GSM2694224 | Terminal Ileum | sporadic colon cancer | 63 | F | Non-involved area | normal tissue | Surgical resection |
| GSM2694225 | Rectum | sporadic colon cancer | 52 | M | Non-involved area | normal tissue | Surgical resection |
| GSM2694226 | Terminal Ileum | sporadic colon cancer | na | na | Non-involved area | normal tissue | Surgical resection |
| GSM2694227 | Terminal Ileum | sporadic colon cancer | 27 | M | Non-involved area | normal tissue | Surgical resection |
| GSM2694228 | Terminal Ileum | Crohn's disease | 44 | F | Inflamed area | Moderate to severe | Surgical resection |
| GSM2694229 | Terminal Ileum | Crohn's disease | 45 | F | Inflamed area | Moderate to severe | Surgical resection |
| GSM2694230 | Rectum | Crohn's disease | 36 | F | Inflamed area | Moderate to severe | Surgical resection |
| GSM2694231 | Terminal Ileum | Crohn's disease | 20 | F | Inflamed area | Moderate to severe | Surgical resection |
| GSM2694232 | Ascending colon | sporadic colon cancer | 20 | F | Non-involved area | normal tissue | Surgical resection |
| GSM2694233 | Terminal Ileum | Crohn's disease | 20 | F | Inflamed area | Moderate to severe | Surgical resection |
| GSM2694234 | Terminal Ileum | Crohn's disease | 41 | F | Inflamed area | Moderate to severe | Surgical resection |
| GSM2694235 | Terminal Ileum | sporadic colon cancer | 39 | F | Non-involved area | normal tissue | Surgical resection |
| GSM2694236 | Terminal Ileum | Crohn's disease | 38 | M | Inflamed area | Moderate to severe | Surgical resection |
| GSM2694237 | Rectum | sporadic colon cancer | 51 | F | Non-involved area | normal tissue | Surgical resection |
| GSM2694238 | Rectum | sporadic colon cancer | 44 | F | Non-involved area | normal tissue | Surgical resection |
| GSM2694239 | Rectum | sporadic colon cancer | 45 | F | Non-involved area | normal tissue | Surgical resection |
| GSM2694240 | Rectum | sporadic colon cancer | na | na | Non-involved area | normal tissue | Surgical resection |
| GSM2694241 | Terminal Ileum | Crohn's disease | 38 | M | Inflamed area | Moderate to severe | Surgical resection |
| GSM2694242 | Rectum | sporadic colon cancer | 26 | M | Non-involved area | normal tissue | Surgical resection |
| GSM2694243 | Descending colon | Crohn's disease | 55 | M | Inflamed area | Moderate to severe | Surgical resection |
| GSM2694244 | Rectum | Crohn's disease | 27 | M | Inflamed area | Moderate to severe | Surgical resection |
| GSM2694245 | Terminal Ileum | sporadic colon cancer | 39 | F | Non-involved area | normal tissue | Surgical resection |
| GSM2694246 | Rectum | Crohn's disease | 20 | M | Inflamed area | Moderate to severe | Surgical resection |
| GSM2694247 | Terminal Ileum | Crohn's disease | 34 | F | Inflamed area | Moderate to severe | Surgical resection |
| GSM2694248 | Terminal Ileum | sporadic colon cancer | 28 | F | Non-involved area | normal tissue | Surgical resection |
| GSM2694249 | Terminal Ileum | Crohn's disease | 40 | M | Inflamed area | Moderate to severe | Surgical resection |
| GSM2694250 | Terminal Ileum | Crohn's disease | 31 | M | Inflamed area | Moderate to severe | Surgical resection |
| GSM2694251 | Sigmoid colon | sporadic colon cancer | 26 | F | Non-involved area | normal tissue | Surgical resection |
| GSM2694252 | Rectum | sporadic colon cancer | 31 | M | Non-involved area | normal tissue | Surgical resection |
| GSM2694253 | Rectum | sporadic colon cancer | 42 | M | Non-involved area | normal tissue | Surgical resection |
| GSM2694254 | Ascending colon | Crohn's disease | 39 | F | Inflamed area | Moderate to severe | Surgical resection |
| GSM2694255 | Rectum | Crohn's disease | 33 | M | Inflamed area | Moderate to severe | Surgical resection |
| GSM2694256 | Sigmoid colon | Crohn's disease | 38 | F | Inflamed area | Moderate to severe | Surgical resection |
| GSM2694257 | Terminal Ileum | sporadic colon cancer | na | na | Non-involved area | normal tissue | Surgical resection |
| GSM2694258 | Terminal Ileum | sporadic colon cancer | 35 | M | Non-involved area | normal tissue | Surgical resection |
| GSM2694259 | Rectum | Crohn's disease | 20 | F | Inflamed area | Moderate to severe | Surgical resection |
| GSM2694260 | Transverse colon | Crohn's disease | 36 | F | Inflamed area | Moderate to severe | Surgical resection |
| GSM2694261 | Descending colon | Crohn's disease | 20 | F | Inflamed area | Moderate to severe | Surgical resection |
| GSM2694262 | Descending colon | Crohn's disease | 20 | F | Inflamed area | Moderate to severe | Surgical resection |
| GSM2694263 | Descending colon | Crohn's disease | 39 | F | Inflamed area | Moderate to severe | Surgical resection |
| GSM2694264 | Sigmoid colon | sporadic colon cancer | 38 | M | Non-involved area | normal tissue | Surgical resection |
| GSM2694265 | Terminal Ileum | sporadic colon cancer | 60 | F | Non-involved area | normal tissue | Surgical resection |
| GSM2694266 | Rectum | Crohn's disease | 55 | M | Inflamed area | Moderate to severe | Surgical resection |
| GSM2694267 | Transverse colon | sporadic colon cancer | 51 | F | Non-involved area | normal tissue | Surgical resection |
| GSM2694268 | Terminal Ileum | Crohn's disease | 29 | F | Inflamed area | Moderate to severe | Surgical resection |
| GSM2694269 | Transverse colon | Crohn's disease | 45 | F | Inflamed area | Moderate to severe | Surgical resection |
| GSM2694270 | Transverse colon | sporadic colon cancer | na | na | Non-involved area | normal tissue | Surgical resection |
| GSM2694271 | Transverse colon | Crohn's disease | 26 | M | Inflamed area | Moderate to severe | Surgical resection |
| GSM2694272 | Transverse colon | sporadic colon cancer | 27 | M | Non-involved area | normal tissue | Surgical resection |
| GSM2694273 | Transverse colon | sporadic colon cancer | 44 | F | Non-involved area | normal tissue | Surgical resection |
| GSM2694274 | Transverse colon | Crohn's disease | 20 | M | Inflamed area | Moderate to severe | Surgical resection |
| GSM2694275 | Descending colon | sporadic colon cancer | 34 | F | Non-involved area | normal tissue | Surgical resection |
| GSM2694276 | Ascending colon | Crohn's disease | 36 | F | Inflamed area | Moderate to severe | Surgical resection |
| GSM2694277 | Transverse colon | sporadic colon cancer | 26 | F | Non-involved area | normal tissue | Surgical resection |
| GSM2694278 | Sigmoid colon | sporadic colon cancer | 31 | M | Non-involved area | normal tissue | Surgical resection |
| GSM2694279 | Descending colon | Crohn's disease | 39 | F | Inflamed area | Moderate to severe | Surgical resection |
| GSM2694280 | Rectum | sporadic colon cancer | 36 | M | Non-involved area | normal tissue | Surgical resection |
| GSM2694281 | Transverse colon | sporadic colon cancer | 38 | F | Non-involved area | normal tissue | Surgical resection |
| GSM2694282 | Transverse colon | Crohn's disease | 42 | M | Inflamed area | Moderate to severe | Surgical resection |
| GSM2694283 | Transverse colon | Crohn's disease | 33 | M | Inflamed area | Moderate to severe | Surgical resection |
| GSM2694284 | Descending colon | Crohn's disease | 41 | F | Inflamed area | Moderate to severe | Surgical resection |
| GSM2694285 | Sigmoid colon | sporadic colon cancer | na | na | Non-involved area | normal tissue | Surgical resection |
| GSM2694286 | Sigmoid colon | sporadic colon cancer | 35 | M | Non-involved area | normal tissue | Surgical resection |
| GSM2694287 | Transverse colon | sporadic colon cancer | na | na | Non-involved area | normal tissue | Surgical resection |
| GSM2694288 | Transverse colon | Crohn's disease | 31 | M | Inflamed area | Moderate to severe | Surgical resection |
| GSM2694289 | Descending colon | sporadic colon cancer | 24 | F | Non-involved area | normal tissue | Surgical resection |
| GSM2694290 | Transverse colon | sporadic colon cancer | 24 | F | Non-involved area | normal tissue | Surgical resection |
| GSM2694291 | Rectum | sporadic colon cancer | 40 | F | Non-involved area | normal tissue | Surgical resection |
| GSM2694292 | Terminal Ileum | Crohn's disease | 51 | F | Inflamed area | Moderate to severe | Surgical resection |
| GSM2694293 | Sigmoid colon | sporadic colon cancer | 51 | F | Non-involved area | normal tissue | Surgical resection |
| GSM2694294 | Sigmoid colon | sporadic colon cancer | 26 | F | Non-involved area | normal tissue | Surgical resection |
| GSM2694295 | Rectum | sporadic colon cancer | 34 | M | Non-involved area | normal tissue | Surgical resection |
| GSM2694296 | Terminal Ileum | sporadic colon cancer | 38 | M | Non-involved area | normal tissue | Surgical resection |
| GSM2694297 | Ascending colon | sporadic colon cancer | 52 | F | Non-involved area | normal tissue | Surgical resection |
| GSM2694298 | Transverse colon | sporadic colon cancer | 41 | F | Non-involved area | normal tissue | Surgical resection |
| GSM2694299 | Transverse colon | Crohn's disease | 48 | M | Inflamed area | Moderate to severe | Surgical resection |
| GSM2694300 | Ascending colon | sporadic colon cancer | 31 | M | Non-involved area | normal tissue | Surgical resection |
| GSM2694301 | Transverse colon | Crohn's disease | 35 | M | Inflamed area | Moderate to severe | Surgical resection |
| GSM2694302 | Transverse colon | sporadic colon cancer | 41 | M | Non-involved area | normal tissue | Surgical resection |
| GSM2694303 | Descending colon | Crohn's disease | 32 | F | Inflamed area | Moderate to severe | Surgical resection |
| GSM2694304 | Ascending colon | sporadic colon cancer | 43 | M | Non-involved area | normal tissue | Surgical resection |
| GSM2694305 | Descending colon | Crohn's disease | na | na | Inflamed area | Moderate to severe | Surgical resection |
| GSM2694306 | Transverse colon | sporadic colon cancer | 52 | M | Non-involved area | normal tissue | Surgical resection |
| GSM2694307 | Transverse colon | Crohn's disease | 19 | M | Inflamed area | Moderate to severe | Surgical resection |
| GSM2694308 | Transverse colon | sporadic colon cancer | 63 | F | Non-involved area | normal tissue | Surgical resection |
| GSM2694309 | Ascending colon | Crohn's disease | 48 | M | Inflamed area | Moderate to severe | Surgical resection |
| GSM2694310 | Terminal Ileum | Crohn's disease | 26 | F | Inflamed area | Moderate to severe | Surgical resection |
| GSM2694311 | Ascending colon | sporadic colon cancer | na | na | Non-involved area | normal tissue | Surgical resection |
| GSM2694312 | Transverse colon | sporadic colon cancer | 60 | M | Non-involved area | normal tissue | Surgical resection |
| GSM2694313 | Rectum | Crohn's disease | 21 | F | Inflamed area | Moderate to severe | Surgical resection |
| GSM2694314 | Sigmoid colon | sporadic colon cancer | 32 | M | Non-involved area | normal tissue | Surgical resection |
| GSM2694315 | Transverse colon | sporadic colon cancer | na | na | Non-involved area | normal tissue | Surgical resection |
| GSM2694316 | Transverse colon | sporadic colon cancer | na | na | Non-involved area | normal tissue | Surgical resection |
| GSM2694317 | Rectum | sporadic colon cancer | na | na | Non-involved area | normal tissue | Surgical resection |
| GSM2694318 | Descending colon | sporadic colon cancer | 40 | M | Non-involved area | normal tissue | Surgical resection |
| GSM2694319 | Terminal Ileum | sporadic colon cancer | na | na | Non-involved area | normal tissue | Surgical resection |
| GSM2694320 | Sigmoid colon | sporadic colon cancer | 60 | M | Non-involved area | normal tissue | Surgical resection |
| GSM2694321 | Transverse colon | sporadic colon cancer | 21 | M | Non-involved area | normal tissue | Surgical resection |
| GSM2694322 | Terminal Ileum | Crohn's disease | 21 | F | Inflamed area | Moderate to severe | Surgical resection |
| GSM2694323 | Rectum | Crohn's disease | na | na | Inflamed area | Moderate to severe | Surgical resection |
| GSM2694324 | Sigmoid colon | sporadic colon cancer | na | na | Non-involved area | normal tissue | Surgical resection |
| GSM2694325 | Rectum | sporadic colon cancer | na | na | Non-involved area | normal tissue | Surgical resection |
| GSM2694326 | Rectum | Crohn's disease | 32 | M | Inflamed area | Moderate to severe | Surgical resection |
| GSM2694327 | Rectum | Crohn's disease | 24 | F | Inflamed area | Moderate to severe | Surgical resection |
| GSM2694328 | Terminal Ileum | Crohn's disease | 40 | F | Inflamed area | Moderate to severe | Surgical resection |
| GSM2694329 | Terminal Ileum | Crohn's disease | 34 | M | Inflamed area | Moderate to severe | Surgical resection |
| GSM2694330 | Rectum | sporadic colon cancer | 26 | F | Non-involved area | normal tissue | Surgical resection |
| GSM2694331 | Rectum | sporadic colon cancer | 24 | F | Non-involved area | normal tissue | Surgical resection |
| GSM2694332 | Rectum | Crohn's disease | 34 | M | Inflamed area | Moderate to severe | Surgical resection |
| GSM2694333 | Ascending colon | Crohn's disease | 27 | M | Inflamed area | Moderate to severe | Surgical resection |
| GSM2694334 | Descending colon | sporadic colon cancer | 27 | M | Non-involved area | normal tissue | Surgical resection |
| GSM2694335 | Descending colon | sporadic colon cancer | 44 | F | Non-involved area | normal tissue | Surgical resection |
| GSM2694336 | Sigmoid colon | sporadic colon cancer | 45 | F | Non-involved area | normal tissue | Surgical resection |
| GSM2694337 | Descending colon | sporadic colon cancer | 51 | F | Non-involved area | normal tissue | Surgical resection |
| GSM2694338 | Ascending colon | sporadic colon cancer | 40 | M | Non-involved area | normal tissue | Surgical resection |
| GSM2694339 | Sigmoid colon | sporadic colon cancer | 26 | M | Non-involved area | normal tissue | Surgical resection |
| GSM2694340 | Descending colon | sporadic colon cancer | na | na | Non-involved area | normal tissue | Surgical resection |
| GSM2694341 | Rectum | sporadic colon cancer | na | na | Non-involved area | normal tissue | Surgical resection |
| GSM2694342 | Sigmoid colon | Crohn's disease | 52 | M | Inflamed area | Moderate to severe | Surgical resection |
| GSM2694343 | Ascending colon | sporadic colon cancer | 34 | M | Non-involved area | normal tissue | Surgical resection |
| GSM2694344 | Ascending colon | sporadic colon cancer | 65 | M | Non-involved area | normal tissue | Surgical resection |
| GSM2694345 | Rectum | Crohn's disease | 19 | M | Inflamed area | Moderate to severe | Surgical resection |
| GSM2694346 | Descending colon | sporadic colon cancer | 63 | F | Non-involved area | normal tissue | Surgical resection |
| GSM2694347 | Rectum | Crohn's disease | 43 | M | Inflamed area | Moderate to severe | Surgical resection |
| GSM2694348 | Descending colon | sporadic colon cancer | na | na | Non-involved area | normal tissue | Surgical resection |
| GSM2694349 | Descending colon | Crohn's disease | 48 | M | Inflamed area | Moderate to severe | Surgical resection |
| GSM2694350 | Transverse colon | Crohn's disease | 51 | F | Inflamed area | Moderate to severe | Surgical resection |
| GSM2694351 | Rectum | sporadic colon cancer | 34 | M | Non-involved area | normal tissue | Surgical resection |
| GSM2694352 | Rectum | Crohn's disease | 24 | M | Inflamed area | Moderate to severe | Surgical resection |
| GSM2694353 | Descending colon | sporadic colon cancer | 65 | M | Non-involved area | normal tissue | Surgical resection |
| GSM2694354 | Transverse colon | Crohn's disease | 34 | M | Inflamed area | Moderate to severe | Surgical resection |
| GSM2694355 | Descending colon | Crohn's disease | 52 | F | Inflamed area | Moderate to severe | Surgical resection |
| GSM2694356 | Rectum | Crohn's disease | 60 | F | Inflamed area | Moderate to severe | Surgical resection |
| GSM2694357 | Rectum | sporadic colon cancer | 35 | F | Non-involved area | normal tissue | Surgical resection |
| GSM2694358 | Sigmoid colon | Crohn's disease | 28 | F | Inflamed area | Moderate to severe | Surgical resection |
| GSM2694359 | Terminal Ileum | Crohn's disease | 34 | M | Inflamed area | Moderate to severe | Surgical resection |
| GSM2694360 | Ascending colon | sporadic colon cancer | 21 | M | Non-involved area | normal tissue | Surgical resection |
| GSM2694362 | Terminal Ileum | sporadic colon cancer | 36 | F | Non-involved area | normal tissue | Surgical resection |
| GSM2694363 | Rectum | Crohn's disease | 48 | F | Inflamed area | Moderate to severe | Surgical resection |
| GSM2694364 | Terminal Ileum | Crohn's disease | 76 | F | Inflamed area | Moderate to severe | Surgical resection |
| GSM2694365 | Transverse colon | sporadic colon cancer | 27 | F | Non-involved area | normal tissue | Surgical resection |
| GSM2694366 | Sigmoid colon | Crohn's disease | 35 | M | Inflamed area | Moderate to severe | Surgical resection |
| GSM2694367 | Descending colon | sporadic colon cancer | 41 | M | Non-involved area | normal tissue | Surgical resection |
| GSM2694368 | Rectum | Crohn's disease | 32 | F | Inflamed area | Moderate to severe | Surgical resection |
| GSM2694369 | Descending colon | sporadic colon cancer | 27 | F | Non-involved area | normal tissue | Surgical resection |
| GSM2694370 | Sigmoid colon | sporadic colon cancer | 65 | M | Non-involved area | normal tissue | Surgical resection |
| GSM2694371 | Descending colon | Crohn's disease | 27 | M | Inflamed area | Moderate to severe | Surgical resection |
| GSM2694372 | Sigmoid colon | sporadic colon cancer | 35 | F | Non-involved area | normal tissue | Surgical resection |
| GSM2694373 | Transverse colon | sporadic colon cancer | 35 | F | Non-involved area | normal tissue | Surgical resection |
| GSM2694374 | Descending colon | Crohn's disease | 55 | F | Inflamed area | Moderate to severe | Surgical resection |
| GSM2694375 | Rectum | Crohn's disease | 57 | M | Inflamed area | Moderate to severe | Surgical resection |
| GSM2694376 | Transverse colon | Crohn's disease | 55 | F | Inflamed area | Moderate to severe | Surgical resection |
| GSM2694377 | Terminal Ileum | sporadic colon cancer | 31 | M | Non-involved area | normal tissue | Surgical resection |
| GSM2694378 | Ascending colon | sporadic colon cancer | na | na | Non-involved area | normal tissue | Surgical resection |
| GSM2694379 | Descending colon | Crohn's disease | 43 | M | Inflamed area | Moderate to severe | Surgical resection |
| GSM2694380 | Ascending colon | sporadic colon cancer | na | na | Non-involved area | normal tissue | Surgical resection |
| GSM2694381 | Sigmoid colon | Crohn's disease | na | na | Inflamed area | Moderate to severe | Surgical resection |
| GSM2694382 | Ascending colon | sporadic colon cancer | 29 | F | Non-involved area | normal tissue | Surgical resection |
| GSM2694383 | Terminal Ileum | Crohn's disease | 35 | F | Inflamed area | Moderate to severe | Surgical resection |
| GSM2694384 | Ascending colon | Crohn's disease | 63 | F | Inflamed area | Moderate to severe | Surgical resection |
| GSM2694385 | Ascending colon | sporadic colon cancer | na | na | Non-involved area | normal tissue | Surgical resection |
| GSM2694386 | Ascending colon | sporadic colon cancer | 29 | M | Non-involved area | normal tissue | Surgical resection |
| GSM2694387 | Descending colon | Crohn's disease | 57 | M | Inflamed area | Moderate to severe | Surgical resection |
| GSM2694388 | Terminal Ileum | Crohn's disease | 29 | M | Inflamed area | Moderate to severe | Surgical resection |
| GSM2694389 | Ascending colon | Crohn's disease | 29 | M | Inflamed area | Moderate to severe | Surgical resection |
| GSM2694390 | Sigmoid colon | Crohn's disease | 31 | F | Inflamed area | Moderate to severe | Surgical resection |
| GSM2694391 | Terminal Ileum | Crohn's disease | 26 | F | Inflamed area | Moderate to severe | Surgical resection |
| GSM2694392 | Rectum | Crohn's disease | 31 | F | Inflamed area | Moderate to severe | Surgical resection |
| GSM2694393 | Rectum | Crohn's disease | 29 | M | Inflamed area | Moderate to severe | Surgical resection |
| GSM2694394 | Sigmoid colon | Crohn's disease | 42 | F | Inflamed area | Moderate to severe | Surgical resection |
| GSM2694395 | Rectum | sporadic colon cancer | 42 | F | Non-involved area | normal tissue | Surgical resection |
| GSM2694396 | Rectum | Crohn's disease | 32 | M | Inflamed area | Moderate to severe | Surgical resection |
| GSM2694397 | Terminal Ileum | sporadic colon cancer | 42 | F | Non-involved area | normal tissue | Surgical resection |
| GSM2694398 | Terminal Ileum | sporadic colon cancer | 36 | F | Non-involved area | normal tissue | Surgical resection |
| GSM2694399 | Ascending colon | sporadic colon cancer | 42 | F | Non-involved area | normal tissue | Surgical resection |
| GSM2694400 | Transverse colon | Crohn's disease | 42 | F | Inflamed area | Moderate to severe | Surgical resection |
| GSM2694401 | Sigmoid colon | Crohn's disease | 46 | F | Inflamed area | Moderate to severe | Surgical resection |
| GSM2694402 | Rectum | Crohn's disease | 46 | F | Inflamed area | Moderate to severe | Surgical resection |
| GSM2694403 | Terminal Ileum | sporadic colon cancer | 31 | F | Non-involved area | normal tissue | Surgical resection |
| GSM2694404 | Terminal Ileum | Crohn's disease | 46 | F | Inflamed area | Moderate to severe | Surgical resection |
| GSM2694405 | Ascending colon | sporadic colon cancer | 46 | F | Non-involved area | normal tissue | Surgical resection |
| GSM2694406 | Transverse colon | sporadic colon cancer | 46 | F | Non-involved area | normal tissue | Surgical resection |
| GSM2694407 | Terminal Ileum | Crohn's disease | 36 | F | Inflamed area | Moderate to severe | Surgical resection |
| GSM2694408 | Rectum | Crohn's disease | 36 | F | Inflamed area | Moderate to severe | Surgical resection |
| GSM2694409 | Ascending colon | sporadic colon cancer | 33 | M | Non-involved area | normal tissue | Surgical resection |
| GSM2694410 | Sigmoid colon | Crohn's disease | 48 | F | Inflamed area | Moderate to severe | Surgical resection |
| GSM2694411 | Sigmoid colon | Crohn's disease | 31 | F | Inflamed area | Moderate to severe | Surgical resection |
| GSM2694412 | Terminal Ileum | Crohn's disease | na | na | Inflamed area | Moderate to severe | Surgical resection |
| GSM2694413 | Descending colon | sporadic colon cancer | na | na | Non-involved area | normal tissue | Surgical resection |
| GSM2694414 | Rectum | Crohn's disease | na | na | Inflamed area | Moderate to severe | Surgical resection |
| GSM2694415 | Transverse colon | sporadic colon cancer | 40 | F | Non-involved area | normal tissue | Surgical resection |
| GSM2694416 | Terminal Ileum | Crohn's disease | 20 | M | Inflamed area | Moderate to severe | Surgical resection |
| GSM2694417 | Terminal Ileum | Crohn's disease | 26 | F | Inflamed area | Moderate to severe | Surgical resection |
| GSM2694418 | Terminal Ileum | Crohn's disease | 38 | F | Inflamed area | Moderate to severe | Surgical resection |
| GSM2694419 | Rectum | sporadic colon cancer | 31 | F | Non-involved area | normal tissue | Surgical resection |
| GSM2694420 | Terminal Ileum | sporadic colon cancer | 31 | F | Non-involved area | normal tissue | Surgical resection |
| GSM2694421 | Rectum | sporadic colon cancer | 76 | F | Non-involved area | normal tissue | Surgical resection |
| GSM2694422 | Transverse colon | sporadic colon cancer | 31 | F | Non-involved area | normal tissue | Surgical resection |
| GSM2694423 | Terminal Ileum | Crohn's disease | 33 | F | Inflamed area | Moderate to severe | Surgical resection |
| GSM2694424 | Ascending colon | Crohn's disease | 33 | F | Inflamed area | Moderate to severe | Surgical resection |
| GSM2694425 | Transverse colon | sporadic colon cancer | 33 | F | Non-involved area | normal tissue | Surgical resection |
| GSM2694426 | Terminal Ileum | Crohn's disease | 20 | M | Inflamed area | Moderate to severe | Surgical resection |
| GSM2694427 | Transverse colon | sporadic colon cancer | 20 | M | Non-involved area | normal tissue | Surgical resection |
| GSM2694428 | Rectum | sporadic colon cancer | 20 | M | Non-involved area | normal tissue | Surgical resection |
| GSM2694429 | Rectum | sporadic colon cancer | 33 | F | Non-involved area | normal tissue | Surgical resection |
| GSM2694430 | Descending colon | sporadic colon cancer | 54 | F | Non-involved area | normal tissue | Surgical resection |
| GSM2694431 | Rectum | sporadic colon cancer | 54 | F | Non-involved area | normal tissue | Surgical resection |
| GSM2694432 | Transverse colon | sporadic colon cancer | 65 | M | Non-involved area | normal tissue | Surgical resection |
| GSM2694433 | Ascending colon | Crohn's disease | na | na | Inflamed area | Moderate to severe | Surgical resection |
| GSM2694434 | Ascending colon | sporadic colon cancer | 54 | F | Non-involved area | normal tissue | Surgical resection |
| GSM2694435 | Ascending colon | Crohn's disease | 24 | F | Inflamed area | Moderate to severe | Surgical resection |
| GSM2694436 | Transverse colon | sporadic colon cancer | 54 | F | Non-involved area | normal tissue | Surgical resection |
| GSM2694437 | Transverse colon | sporadic colon cancer | na | na | Non-involved area | normal tissue | Surgical resection |
| GSM2694438 | Ascending colon | Crohn's disease | na | na | Inflamed area | Moderate to severe | Surgical resection |
| GSM2694439 | Terminal Ileum | Crohn's disease | 34 | F | Inflamed area | Moderate to severe | Surgical resection |
| GSM2694440 | Ascending colon | Crohn's disease | 32 | M | Inflamed area | Moderate to severe | Surgical resection |
| GSM2694441 | Ascending colon | sporadic colon cancer | 60 | M | Non-involved area | normal tissue | Surgical resection |
| GSM2694442 | Ascending colon | sporadic colon cancer | 21 | F | Non-involved area | normal tissue | Surgical resection |
| GSM2694443 | Transverse colon | Crohn's disease | 27 | M | Inflamed area | Moderate to severe | Surgical resection |
| GSM2694444 | Descending colon | sporadic colon cancer | 35 | F | Non-involved area | normal tissue | Surgical resection |
| GSM2694445 | Transverse colon | sporadic colon cancer | 65 | M | Non-involved area | normal tissue | Surgical resection |
| GSM2694446 | Descending colon | Crohn's disease | 24 | M | Inflamed area | Moderate to severe | Surgical resection |
| GSM2694447 | Ascending colon | Crohn's disease | 26 | F | Inflamed area | Moderate to severe | Surgical resection |
| GSM2694448 | Transverse colon | Crohn's disease | 39 | F | Inflamed area | Moderate to severe | Surgical resection |
| GSM2694449 | Transverse colon | sporadic colon cancer | 40 | M | Non-involved area | normal tissue | Surgical resection |
| GSM2694450 | Ascending colon | Crohn's disease | 35 | M | Inflamed area | Moderate to severe | Surgical resection |
| GSM2694451 | Ascending colon | sporadic colon cancer | 56 | M | Non-involved area | normal tissue | Surgical resection |
| GSM2694452 | Descending colon | sporadic colon cancer | 56 | M | Non-involved area | normal tissue | Surgical resection |
| GSM2694453 | Rectum | sporadic colon cancer | 56 | M | Non-involved area | normal tissue | Surgical resection |
| GSM2694454 | Terminal Ileum | Crohn's disease | 56 | M | Inflamed area | Moderate to severe | Surgical resection |
| GSM2694455 | Transverse colon | sporadic colon cancer | 56 | M | Non-involved area | normal tissue | Surgical resection |
| GSM2694456 | Ascending colon | Crohn's disease | 20 | M | Inflamed area | Moderate to severe | Surgical resection |
| GSM2694457 | Transverse colon | sporadic colon cancer | 36 | M | Non-involved area | normal tissue | Surgical resection |
| GSM2694458 | Sigmoid colon | Crohn's disease | 60 | F | Inflamed area | Moderate to severe | Surgical resection |
| GSM2694459 | Transverse colon | Crohn's disease | 26 | F | Inflamed area | Moderate to severe | Surgical resection |
| GSM2694460 | Rectum | Crohn's disease | 26 | F | Inflamed area | Moderate to severe | Surgical resection |
| GSM2694461 | Ascending colon | sporadic colon cancer | 26 | F | Non-involved area | normal tissue | Surgical resection |
| GSM2694462 | Rectum | Crohn's disease | 26 | F | Inflamed area | Moderate to severe | Surgical resection |
| GSM2694463 | Transverse colon | sporadic colon cancer | 44 | F | Non-involved area | normal tissue | Surgical resection |
| GSM2694464 | Rectum | sporadic colon cancer | 42 | M | Non-involved area | normal tissue | Surgical resection |
| GSM2694465 | Terminal Ileum | Crohn's disease | 42 | M | Inflamed area | Moderate to severe | Surgical resection |
| GSM2694466 | Descending colon | sporadic colon cancer | 42 | M | Non-involved area | normal tissue | Surgical resection |
| GSM2694467 | Sigmoid colon | sporadic colon cancer | 42 | M | Non-involved area | normal tissue | Surgical resection |
| GSM2694468 | Descending colon | Crohn's disease | 28 | F | Inflamed area | Moderate to severe | Surgical resection |
| GSM2694469 | Sigmoid colon | sporadic colon cancer | 35 | F | Non-involved area | normal tissue | Surgical resection |
| GSM2694470 | Sigmoid colon | Crohn's disease | 34 | F | Inflamed area | Moderate to severe | Surgical resection |
| GSM2694471 | Rectum | Crohn's disease | 55 | F | Inflamed area | Moderate to severe | Surgical resection |
| GSM2694472 | Terminal Ileum | Crohn's disease | 44 | F | Inflamed area | Moderate to severe | Surgical resection |
| GSM2694473 | Rectum | sporadic colon cancer | 65 | M | Non-involved area | normal tissue | Surgical resection |
| GSM2694474 | Rectum | sporadic colon cancer | 27 | M | Non-involved area | normal tissue | Surgical resection |
| GSM2694475 | Ascending colon | sporadic colon cancer | 32 | F | Non-involved area | normal tissue | Surgical resection |
| GSM2694476 | Transverse colon | sporadic colon cancer | 48 | F | Non-involved area | normal tissue | Surgical resection |
| GSM2694477 | Terminal Ileum | sporadic colon cancer | 48 | F | Non-involved area | normal tissue | Surgical resection |
| GSM2694478 | Rectum | sporadic colon cancer | 35 | F | Non-involved area | normal tissue | Surgical resection |
| GSM2694479 | Transverse colon | sporadic colon cancer | 76 | F | Non-involved area | normal tissue | Surgical resection |
| GSM2694480 | Rectum | Crohn's disease | 48 | M | Inflamed area | Moderate to severe | Surgical resection |
| GSM2694481 | Descending colon | Crohn's disease | 51 | F | Inflamed area | Moderate to severe | Surgical resection |
| GSM2694482 | Terminal Ileum | Crohn's disease | 34 | M | Inflamed area | Moderate to severe | Surgical resection |
| GSM2694483 | Ascending colon | Crohn's disease | 34 | F | Inflamed area | Moderate to severe | Surgical resection |
| GSM2694484 | Terminal Ileum | Crohn's disease | 24 | M | Inflamed area | Moderate to severe | Surgical resection |
| GSM2694485 | Rectum | sporadic colon cancer | 65 | M | Non-involved area | normal tissue | Surgical resection |
| GSM2694486 | Transverse colon | sporadic colon cancer | na | na | Non-involved area | normal tissue | Surgical resection |
| GSM2694487 | Rectum | sporadic colon cancer | na | na | Non-involved area | normal tissue | Surgical resection |
| GSM2694488 | Rectum | sporadic colon cancer | 60 | M | Non-involved area | normal tissue | Surgical resection |
| GSM2694489 | Terminal Ileum | Crohn's disease | 43 | M | Inflamed area | Moderate to severe | Surgical resection |
| GSM2694490 | Terminal Ileum | sporadic colon cancer | na | na | Non-involved area | normal tissue | Surgical resection |
| GSM2694491 | Sigmoid colon | Crohn's disease | 52 | M | Inflamed area | Moderate to severe | Surgical resection |
| GSM2694492 | Terminal Ileum | Crohn's disease | 32 | M | Inflamed area | Moderate to severe | Surgical resection |
| GSM2694493 | Terminal Ileum | sporadic colon cancer | 24 | F | Non-involved area | normal tissue | Surgical resection |
| GSM2694494 | Ascending colon | Crohn's disease | na | na | Inflamed area | Moderate to severe | Surgical resection |
| GSM2694495 | Rectum | Crohn's disease | 35 | M | Inflamed area | Moderate to severe | Surgical resection |
| GSM2694496 | Rectum | Crohn's disease | 41 | M | Inflamed area | Moderate to severe | Surgical resection |
| GSM2694497 | Terminal Ileum | Crohn's disease | 51 | F | Inflamed area | Moderate to severe | Surgical resection |
| GSM2694498 | Terminal Ileum | sporadic colon cancer | 26 | F | Non-involved area | normal tissue | Surgical resection |
| GSM2694499 | Terminal Ileum | Crohn's disease | 24 | F | Inflamed area | Moderate to severe | Surgical resection |
| GSM2694500 | Descending colon | Crohn's disease | 42 | M | Inflamed area | Moderate to severe | Surgical resection |
| GSM2694501 | Ascending colon | sporadic colon cancer | 55 | F | Non-involved area | normal tissue | Surgical resection |
| GSM2694502 | Sigmoid colon | Crohn's disease | 33 | M | Inflamed area | Moderate to severe | Surgical resection |
| GSM2694503 | Rectum | Crohn's disease | na | na | Inflamed area | Moderate to severe | Surgical resection |
| GSM2694504 | Rectum | sporadic colon cancer | 35 | M | Non-involved area | normal tissue | Surgical resection |
| GSM2694506 | Descending colon | Crohn's disease | 31 | M | Inflamed area | Moderate to severe | Surgical resection |
| GSM2694507 | Rectum | sporadic colon cancer | 41 | F | Non-involved area | normal tissue | Surgical resection |
| GSM2694508 | Descending colon | Crohn's disease | 36 | F | Inflamed area | Moderate to severe | Surgical resection |
| GSM2694509 | Terminal Ileum | sporadic colon cancer | 71 | F | Non-involved area | normal tissue | Surgical resection |
| GSM2694510 | Rectum | Crohn's disease | 20 | F | Inflamed area | Moderate to severe | Surgical resection |
| GSM2694511 | Rectum | sporadic colon cancer | 39 | F | Non-involved area | normal tissue | Surgical resection |
| GSM2694512 | Rectum | Crohn's disease | 38 | M | Inflamed area | Moderate to severe | Surgical resection |
| GSM2694513 | Terminal Ileum | Crohn's disease | 55 | M | Inflamed area | Moderate to severe | Surgical resection |
| GSM2694514 | Rectum | Crohn's disease | 34 | F | Inflamed area | Moderate to severe | Surgical resection |
| GSM2694515 | Rectum | Crohn's disease | 28 | F | Inflamed area | Moderate to severe | Surgical resection |

F: Female; M: Male; na: not available.

**Supplementary Table 1b. Summary of Crohn’s disease (CD) populations in GSE100833.**

| **Number of individuals** | **Number of samples** | **Disease activity** | **Sex** | **Age Range** | **Age Mean** | **Tissues**  **Profiled** | **Profiling** |
| --- | --- | --- | --- | --- | --- | --- | --- |
| 87 | 159 | Moderate to severe | Female 50%  Male 44%  Non-specified 6% | >18 years | 39 | Terminal ileum 31%  Ascending colon 12%  Transvers colon 11%  Descending colon 14%  Sigmoid colon 9%  Rectum 23% | Microarray |

**Supplementary Table 1C. Clinicopathological characteristics of the included samples in GSE16879 dataset.**

| **Source** | **Tissue /Location** | **Diagnosis** | **inflammation** | **Disease activity** | **Method** |
| --- | --- | --- | --- | --- | --- |
| GSM364627 | colon | control individuals | normal area | normal tissue | endoscopy biopsy |
| GSM364628 | colon | control individuals | normal area | normal tissue | endoscopy biopsy |
| GSM364629 | colon | control individuals | normal area | normal tissue | endoscopy biopsy |
| GSM364630 | colon | control individuals | normal area | normal tissue | endoscopy biopsy |
| GSM364631 | colon | control individuals | normal area | normal tissue | endoscopy biopsy |
| GSM364632 | colon | control individuals | normal area | normal tissue | endoscopy biopsy |
| GSM423010 | colon | Crohn's disease | Inflamed area | active inflammation | endoscopy biopsy |
| GSM423011 | colon | Crohn's disease | Inflamed area | active inflammation | endoscopy biopsy |
| GSM423012 | colon | Crohn's disease | Inflamed area | active inflammation | endoscopy biopsy |
| GSM423013 | colon | Crohn's disease | Inflamed area | active inflammation | endoscopy biopsy |
| GSM423014 | colon | Crohn's disease | Inflamed area | active inflammation | endoscopy biopsy |
| GSM423015 | colon | Crohn's disease | Inflamed area | active inflammation | endoscopy biopsy |
| GSM423016 | colon | Crohn's disease | Inflamed area | active inflammation | endoscopy biopsy |
| GSM423017 | colon | Crohn's disease | Inflamed area | active inflammation | endoscopy biopsy |
| GSM423018 | colon | Crohn's disease | Inflamed area | active inflammation | endoscopy biopsy |
| GSM423019 | colon | Crohn's disease | Inflamed area | active inflammation | endoscopy biopsy |
| GSM423020 | colon | Crohn's disease | Inflamed area | active inflammation | endoscopy biopsy |
| GSM423021 | colon | Crohn's disease | Inflamed area | active inflammation | endoscopy biopsy |
| GSM423022 | colon | Crohn's disease | Inflamed area | active inflammation | endoscopy biopsy |
| GSM423023 | colon | Crohn's disease | Inflamed area | active inflammation | endoscopy biopsy |
| GSM423024 | colon | Crohn's disease | Inflamed area | active inflammation | endoscopy biopsy |
| GSM423025 | colon | Crohn's disease | Inflamed area | active inflammation | endoscopy biopsy |
| GSM423026 | colon | Crohn's disease | Inflamed area | active inflammation | endoscopy biopsy |
| GSM423027 | colon | Crohn's disease | Inflamed area | active inflammation | endoscopy biopsy |
| GSM423028 | colon | Crohn's disease | Inflamed area | active inflammation | endoscopy biopsy |
| GSM423029 | colon | Crohn's disease | Inflamed area | active inflammation | endoscopy biopsy |
| GSM423030 | colon | Crohn's disease | Inflamed area | active inflammation | endoscopy biopsy |
| GSM423031 | colon | Crohn's disease | Inflamed area | active inflammation | endoscopy biopsy |
| GSM423032 | colon | Crohn's disease | Inflamed area | active inflammation | endoscopy biopsy |
| GSM423033 | colon | Crohn's disease | Inflamed area | active inflammation | endoscopy biopsy |
| GSM423034 | colon | Crohn's disease | Inflamed area | active inflammation | endoscopy biopsy |
| GSM423035 | colon | Crohn's disease | Inflamed area | active inflammation | endoscopy biopsy |
| GSM423036 | colon | Crohn's disease | Inflamed area | active inflammation | endoscopy biopsy |
| GSM423037 | colon | Crohn's disease | Inflamed area | active inflammation | endoscopy biopsy |
| GSM423038 | colon | Crohn's disease | Inflamed area | active inflammation | endoscopy biopsy |
| GSM423039 | colon | Crohn's disease | Inflamed area | active inflammation | endoscopy biopsy |
| GSM423040 | colon | Crohn's disease | Inflamed area | active inflammation | endoscopy biopsy |
| GSM423041 | colon | Crohn's disease | Inflamed area | active inflammation | endoscopy biopsy |
| GSM423042 | colon | Crohn's disease | Inflamed area | active inflammation | endoscopy biopsy |
| GSM423043 | colon | Crohn's disease | Inflamed area | active inflammation | endoscopy biopsy |
| GSM423044 | colon | Crohn's disease | Inflamed area | active inflammation | endoscopy biopsy |
| GSM423045 | colon | Crohn's disease | Inflamed area | active inflammation | endoscopy biopsy |
| GSM423046 | colon | Crohn's disease | Inflamed area | active inflammation | endoscopy biopsy |
| GSM423047 | Ileum | control individuals | normal area | normal tissue | endoscopy biopsy |
| GSM423048 | Ileum | control individuals | normal area | normal tissue | endoscopy biopsy |
| GSM423049 | Ileum | control individuals | normal area | normal tissue | endoscopy biopsy |
| GSM423050 | Ileum | control individuals | normal area | normal tissue | endoscopy biopsy |
| GSM423051 | Ileum | control individuals | normal area | normal tissue | endoscopy biopsy |
| GSM423052 | Ileum | control individuals | normal area | normal tissue | endoscopy biopsy |
| GSM423053 | Ileum | Crohn's disease | Inflamed area | active inflammation | endoscopy biopsy |
| GSM423054 | Ileum | Crohn's disease | Inflamed area | active inflammation | endoscopy biopsy |
| GSM423055 | Ileum | Crohn's disease | Inflamed area | active inflammation | endoscopy biopsy |
| GSM423056 | Ileum | Crohn's disease | Inflamed area | active inflammation | endoscopy biopsy |
| GSM423057 | Ileum | Crohn's disease | Inflamed area | active inflammation | endoscopy biopsy |
| GSM423058 | Ileum | Crohn's disease | Inflamed area | active inflammation | endoscopy biopsy |
| GSM423059 | Ileum | Crohn's disease | Inflamed area | active inflammation | endoscopy biopsy |
| GSM423060 | Ileum | Crohn's disease | Inflamed area | active inflammation | endoscopy biopsy |
| GSM423061 | Ileum | Crohn's disease | Inflamed area | active inflammation | endoscopy biopsy |
| GSM423062 | Ileum | Crohn's disease | Inflamed area | active inflammation | endoscopy biopsy |
| GSM423063 | Ileum | Crohn's disease | Inflamed area | active inflammation | endoscopy biopsy |
| GSM423064 | Ileum | Crohn's disease | Inflamed area | active inflammation | endoscopy biopsy |
| GSM423065 | Ileum | Crohn's disease | Inflamed area | active inflammation | endoscopy biopsy |
| GSM423066 | Ileum | Crohn's disease | Inflamed area | active inflammation | endoscopy biopsy |
| GSM423067 | Ileum | Crohn's disease | Inflamed area | active inflammation | endoscopy biopsy |
| GSM423068 | Ileum | Crohn's disease | Inflamed area | active inflammation | endoscopy biopsy |
| GSM423069 | Ileum | Crohn's disease | Inflamed area | active inflammation | endoscopy biopsy |
| GSM423070 | Ileum | Crohn's disease | Inflamed area | active inflammation | endoscopy biopsy |
| GSM423071 | Ileum | Crohn's disease | Inflamed area | active inflammation | endoscopy biopsy |
| GSM423072 | Ileum | Crohn's disease | Inflamed area | active inflammation | endoscopy biopsy |
| GSM423073 | Ileum | Crohn's disease | Inflamed area | active inflammation | endoscopy biopsy |
| GSM423074 | Ileum | Crohn's disease | Inflamed area | active inflammation | endoscopy biopsy |
| GSM423075 | Ileum | Crohn's disease | Inflamed area | active inflammation | endoscopy biopsy |
| GSM423076 | Ileum | Crohn's disease | Inflamed area | active inflammation | endoscopy biopsy |
| GSM423077 | Ileum | Crohn's disease | Inflamed area | active inflammation | endoscopy biopsy |
| GSM423078 | Ileum | Crohn's disease | Inflamed area | active inflammation | endoscopy biopsy |
| GSM423079 | Ileum | Crohn's disease | Inflamed area | active inflammation | endoscopy biopsy |
| GSM423080 | Ileum | Crohn's disease | Inflamed area | active inflammation | endoscopy biopsy |
| GSM423081 | Ileum | Crohn's disease | Inflamed area | active inflammation | endoscopy biopsy |
| GSM423082 | Ileum | Crohn's disease | Inflamed area | active inflammation | endoscopy biopsy |
| GSM423083 | Ileum | Crohn's disease | Inflamed area | active inflammation | endoscopy biopsy |
| GSM423084 | Ileum | Crohn's disease | Inflamed area | active inflammation | endoscopy biopsy |
| GSM423085 | Ileum | Crohn's disease | Inflamed area | active inflammation | endoscopy biopsy |
| GSM423086 | Ileum | Crohn's disease | Inflamed area | active inflammation | endoscopy biopsy |
| GSM423087 | Ileum | Crohn's disease | Inflamed area | active inflammation | endoscopy biopsy |
| GSM423088 | Ileum | Crohn's disease | Inflamed area | active inflammation | endoscopy biopsy |

**Supplementary Table 1d. Summary of Crohn’s disease (CD) populations in GSE16879.**

| **Number of individuals** | **Number of samples** | **Disease activity** | **Sex** | **Age Range** | **Age Mean** | **Tissues**  **Profiled** | **Profiling** |
| --- | --- | --- | --- | --- | --- | --- | --- |
| 37 | 73 | Active | Female 46%  Male 54% | >18 years | 39.1 | Colon 51%  Ileum 49% | Microarray |
