# Supplementary material for: Investigating regulatory patterns of NLRP3 Inflammasome features and association with immune microenvironment in Crohn’s disease
Source: Front Immunol. 2023 Jan 5;13:1096587. doi: 10.3389/fimmu.2022.1096587 (PMC9849378; doi:10.3389/fimmu.2022.1096587)
Supplement: Supplementary file 3 [file Table_2.docx]

**Supplementary Table 2. Identification of differentially expressed NLRP3 inflammasome-related genes between diseased and control samples.**

| **Gene** | **logFC** | **AveExpr** | **t** | **P.Value** | **adj.P.Val** | **B** |
| --- | --- | --- | --- | --- | --- | --- |
| GBP5 | -1.45106 | 6.763396 | -12.1884 | 1.87E-28 | 4.68E-27 | 53.76853 |
| CASP1 | -0.84021 | 9.168508 | -10.9539 | 5.14E-24 | 6.43E-23 | 43.63371 |
| CARD8 | -0.41851 | 7.365042 | -8.98703 | 2.03E-17 | 1.69E-16 | 28.61073 |
| TLR4 | -0.54282 | 5.387067 | -7.85641 | 5.66E-14 | 3.54E-13 | 20.78757 |
| PANX1 | -0.54094 | 7.24918 | -7.41636 | 1.03E-12 | 5.15E-12 | 17.93498 |
| NLRP3 | -0.34737 | 3.982414 | -5.9268 | 7.80E-09 | 3.25E-08 | 9.196208 |
| TLR6 | -0.25381 | 4.46892 | -5.81834 | 1.41E-08 | 5.03E-08 | 8.619935 |
| PSTPIP1 | -0.38017 | 5.378591 | -5.18558 | 3.77E-07 | 1.18E-06 | 5.433598 |
| NFKB1 | -0.28901 | 7.724881 | -4.84783 | 1.93E-06 | 5.35E-06 | 3.860122 |
| NFKB2 | -0.17178 | 4.733662 | -4.62292 | 5.44E-06 | 1.36E-05 | 2.86329 |
| HSP90AB1 | -0.2326 | 10.33868 | -4.2499 | 2.79E-05 | 6.34E-05 | 1.302338 |
| NLRC3 | -0.34534 | 7.231991 | -4.0861 | 5.52E-05 | 0.000115 | 0.653964 |
| RELA | -0.13282 | 7.387779 | -3.693 | 0.000259 | 0.000499 | -0.80738 |
| MEFV | -0.11524 | 7.163631 | -2.25971 | 0.024493 | 0.043737 | -4.95492 |
| DHX33 | -0.12267 | 8.785454 | -2.17496 | 0.030344 | 0.050574 | -5.14004 |
| TXN | 0.068291 | 11.47243 | 2.101913 | 0.036321 | 0.056751 | -5.29411 |
| APP | 0.047445 | 10.18899 | 1.745256 | 0.081874 | 0.120402 | -5.97242 |
| SIRT2 | -0.05128 | 4.903937 | -1.67409 | 0.095062 | 0.132031 | -6.09301 |
| SUGT1 | 0.033571 | 8.028122 | 1.187765 | 0.235783 | 0.31024 | -6.78444 |
| P2RX7 | -0.03362 | 3.128736 | -1.06923 | 0.28575 | 0.357187 | -6.91769 |
| EIF2AK2 | -0.02448 | 6.374465 | -0.86766 | 0.386211 | 0.459775 | -7.11232 |
| CD36 | 0.050397 | 6.328233 | 0.754883 | 0.450859 | 0.51234 | -7.20363 |
| TXNIP | 0.005719 | 11.25617 | 0.097439 | 0.922437 | 0.984625 | -7.48355 |
| GSDMD | -0.00365 | 7.61774 | -0.05791 | 0.953853 | 0.984625 | -7.48662 |
| PYCARD | -0.00097 | 8.436102 | -0.01929 | 0.984625 | 0.984625 | -7.48811 |
